# Three Dopamine Pathways Induce Aversive Odor Memories with Different Stability

**DOI:** 10.1371/journal.pgen.1002768

**Published:** 2012-07-12

**Authors:** Yoshinori Aso, Andrea Herb, Maite Ogueta, Igor Siwanowicz, Thomas Templier, Anja B. Friedrich, Kei Ito, Henrike Scholz, Hiromu Tanimoto

**Affiliations:** 1 Max Planck Institut für Neurobiologie, Martinsried, Germany; 2 Lehrstuhl für Genetik und Neurobiologie, Universität Würzburg, Würzburg, Germany; 3 Institute of Molecular and Cellular Biosciences, The University of Tokyo, Tokyo, Japan; 4 Universität zu Köln, Biozentrum Köln, Köln, Germany; University of California San Francisco, United States of America

## Abstract

Animals acquire predictive values of sensory stimuli through reinforcement. In the brain of *Drosophila melanogaster*, activation of two types of dopamine neurons in the PAM and PPL1 clusters has been shown to induce aversive odor memory. Here, we identified the third cell type and characterized aversive memories induced by these dopamine neurons. These three dopamine pathways all project to the mushroom body but terminate in the spatially segregated subdomains. To understand the functional difference of these dopamine pathways in electric shock reinforcement, we blocked each one of them during memory acquisition. We found that all three pathways partially contribute to electric shock memory. Notably, the memories mediated by these neurons differed in temporal stability. Furthermore, combinatorial activation of two of these pathways revealed significant interaction of individual memory components rather than their simple summation. These results cast light on a cellular mechanism by which a noxious event induces different dopamine signals to a single brain structure to synthesize an aversive memory.

## Author Summary

Punishment not only repels animals but also drives the formation of aversive memory of contiguous stimuli. Guided by the memory, animals can later avoid the cues that predict negative outcome. How is a punishing event represented in the brain? We have found that at least three types of dopamine neurons in the *Drosophila* brain contribute to memory formation. Genetic activation of these neurons temporally paired with an odor presentation induced aversive odor memory, raising a question about the functional distinction of these neurons. Here we characterized aversive memories induced by these dopamine neurons. The magnitude of immediate memory and following memory decay differ greatly among the three cell types. Interestingly, combinatorial activation of two cell types revealed that induced memory is not a simple sum of the two memories, but rather the result of non-linear interaction specific for different retention times. Taken together, we propose that a punishing event induces aversive memory with unique temporal dynamics by tuning the activation of selective dopamine neurons.

## Introduction

**Figure 1:**
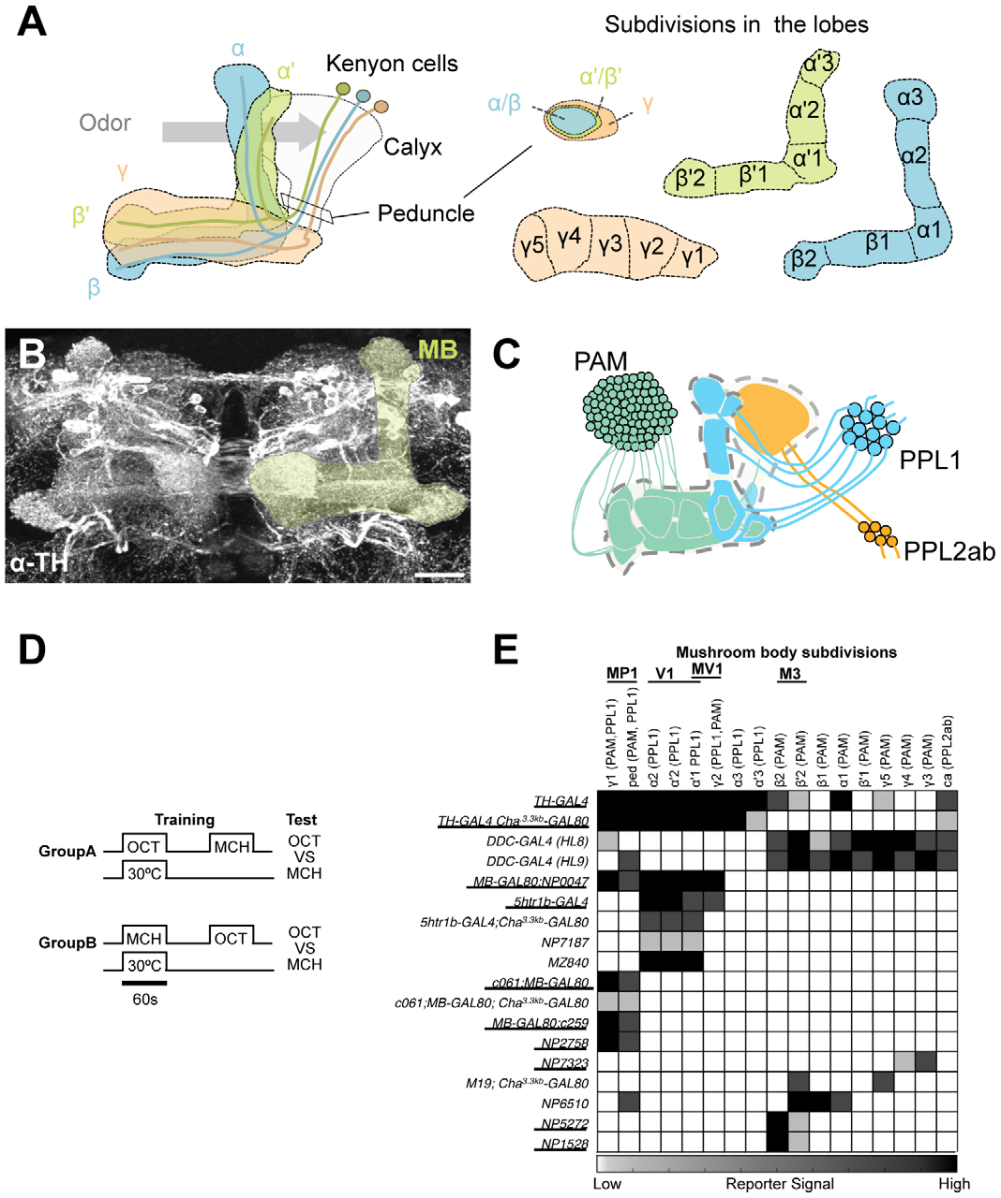
GAL4 drivers for dopaminergic neurons that project to the mushroom body. (A) Schematic diagrams of the mushroom body (left) and subdivisions in the lobe system of the MB (right). Kenyon cells are the major intrinsic neurons of the MB. They have dendritic terminals forming the structure called calyx, where odor signals are conveyed from the antennal lobes. From the calyx, Kenyon cell axons project anteriorly through the peduncle to the lobes. α, α', β, β', and γ indicate the lobes of the MB. In the peduncle, the parallel axon fibers of Kenyon cell subtypes are organized in concentric layers. The spur is contributed exclusively by the neurons projecting to the γ lobe (γ1). The terminals of the extrinsic neurons define the subdivision in the lobes along the longitudinal axis of Kenyon cell axon bundles [15]. (B) Confocal projection of the MB region (rectangle in A; light green overlay) labeled with the antibody against tyrosine hydroxylase (TH; frontal view; dorsal up). Many types of dopaminergic neurons project to the entire MB. Scale bar represents 20 µm. (C) The MB is supplied by three distinct clusters of TH-immunoreactive cells (PAM, PPL1, and PPL2ab) [21], [22]. (D) The conditioning protocol for dTRPA1-induced memory (see Material and Methods for detail). (E) Terminal distribution of dopamine neurons in the MB in GAL4 drivers used in this study. The gray scale represents subjectively determined intensity of terminals in the MB. Drivers that induced significant aversive memory are underlined. See Figure S2 for the fraction of GAL4 expressing cells per cluster. See [21], [22] for the description of the clusters.

Mechanisms underlying memory can be as simple as a modulation of monosynaptic connection in the gill withdraw reflex of *Aplysia* [1]. Alternatively memory formation and storage may require dynamic interaction of distinct neuropiles in a brain [2]. In *Drosophila melanogaster*, a neuronal circuit centered on the mushroom body (MB) is important for the formation and storage of odor memory [3], [4], [5], [6]. Signals of odor and shock are integrated in the MB for memory formation [7], [8], [9]. Identity of the odor is represented by a small subset of Kenyon cells [10], [11], [12], which are major intrinsic neurons of the MB and categorized into the α/β, α'/β' and γ neurons (Figure 1A) [13]. According to their projection patterns in the lobes, these neurons can be further classified into 8 subtypes: α/βp, α/βs and α/βc (also known as the pioneer α/β, the early α/β, or the late α/β respectively [14]) for the posterior, surface or core layers of the α/β lobes; α'/β'a, α'/β'm and α'/β'p for the anterior, middle and posterior layers of α'/β' lobes; γd and γmain for the dorsal and main layer of the γ lobe [15], [16]. The Kenyon cells receive a dopamine signal that mediates aversive reinforcement for odor memory formation [17], [18]. Expression of DopR, a D1-like dopamine receptor also known as dDA1, in the γ neurons is fully sufficient to rescue the mutant defect in aversive odor memory [18]. Activation of many types of dopamine neurons using *TH-GAL4* can substitute for the aversive stimulus that induces odor memory [19], [20]. In flies, dopamine neurons from protocerebral anterior median (PAM), protocerebral posterior lateral 1 and 2ab (PPL1 and PPL2ab) clusters terminate in the entire MB (Figure 1B–1C) [21], [22], [23]. Individual neurons in these clusters terminate distinct subdomains along the longitudinal axis of the MB lobes. The application of noxious stimuli, such as electric shock, activates only a subset of dopamine neurons [21], [24], indicating that the response property greatly varies among individual cells within a cluster. Consistent with this observation, activation of specific subsets of these clusters, such as MB-M3 and MB-MP1 neurons, can induce aversive odor memory [25]. This dopamine input presumably modulates the pre-synaptic output of odor-representing Kenyon cells and drives memory formation [8], [9].

Besides aversive reinforcement, dopamine is responsible for a wide range of physiological functions including appetitive memory, some of which are mapped in the MB [17], [21], [24], [26], [27], [28], [29]. This implies the functional differentiation of the MB subdivisions by different sets of dopamine neurons. While both MB-M3 and MB-MP1 neurons can induce aversive odor memory, these neurons terminate in spatially segregated subdomains of the MB. MB-M3 neurons primarily synapses in the medial tip of the β lobe, whereas MB-MP1 neurons terminates in the spur of the γ lobe (γ1) and the peduncle of the α/β neurons [15]. Blocking the output of MB-M3 neurons during memory acquisition preferentially affects labile 2-hour memory [25], indicating the partial contribution of MB-M3 neurons to aversive reinforcement of electric shock. This raises the question of how the other reinforcement pathways to the MB are coordinated to synthesize a complete aversive odor memory. Considering the selective phenotype of MB-M3 neurons and differential functions of the lobe systems [25], [30], [31], [32], [33], it is likely that electric shock induces qualitatively different memory traces in parallel. In this study, we challenge this hypothesis by activating and inactivating individual dopamine pathways separately. By conducting further behavioral screening of GAL4 driver lines, we identify a third type of dopamine neurons that are capable to induce aversive odor memory. Using these tools to manipulate different dopamine pathways to the MB, we characterize each memory component in isolation and show how their interaction shapes the temporal stability of aversive memory.

## Results

### Screening dopamine neurons for formation of aversive memory

To systematically identify reinforcement pathways to the MB, we elaborated our previous dTRPA1-based behavioral screening by testing 10 additional GAL4 lines for different subsets of dopamine neurons. Dopamine synthesis in MB-projecting neurons of these lines was confirmed with immunoreactive signals to Tyrosine Hydroxylase (TH), the rate-limiting enzyme for dopamine synthesis. The pattern of TH signals coincides well with that of dopamine itself in the brain (Figure S1). This collection of GAL4 drivers covers most, if not all, dopamine neurons in the fly brain including those projecting to the MB (Figure S2) [25]. For activation of target neurons, we expressed the thermo-sensitive cation channel dTRPA1 [34]. We elevated temperature to 30°C for 60 sec only during the presentation of an odor, as in the standard conditioning protocol (Figure 1D). This temperature shift by itself had a slight aversive effect as seen in the tendency of conditioned odor avoidance of control groups [25]. Contingent activation with the odor presentation should induce significant conditioned avoidance of the paired odor, if a given GAL4 line drives dTrpA1 within neurons that signal aversive reinforcement. We found functional heterogeneity of dopamine neurons in inducing immediate aversive memory (Figure 1E). Activation of particular cell types in the PAM and PPL1 clusters induced aversive memory, whereas drivers labeling other types in the same clusters did not (Figure 1E; see below for the detailed description).

However, most GAL4 drivers used in these experiments have expression outside the target dopamine neurons. To scrutinize whether the induced memory is due to the target cells, we suppressed dTRPA1 expression selectively in dopamine neurons using *TH-GAL80* [35] and examined whether *TH-GAL80* suppresses memory formation induced by thermo-activation. We found that aversive memory is indeed attributable to dopamine neurons in most cases. *TH-GAL80* did not fully silence dTRPA1-dependent memory of *MB-GAL80;c259* and *NP2758*, which could be due to either an incomplete suppression of GAL4 in dopamine neurons or a potential contribution of non-dopaminergic cells in these drivers (see below for *MB-GAL80;c259* and [25] for *NP2758*). To further narrow down the expression pattern to a single dopamine cell type, we combined *Cha^3.3kb^-GAL80* with some drivers. The *Cha^3.3kb^* fragment drives strong expression not only in choline acetyltransferase-positive neurons but also non-cholinergic cells presumably due to ectopic expression [36].

**Figure 2:**
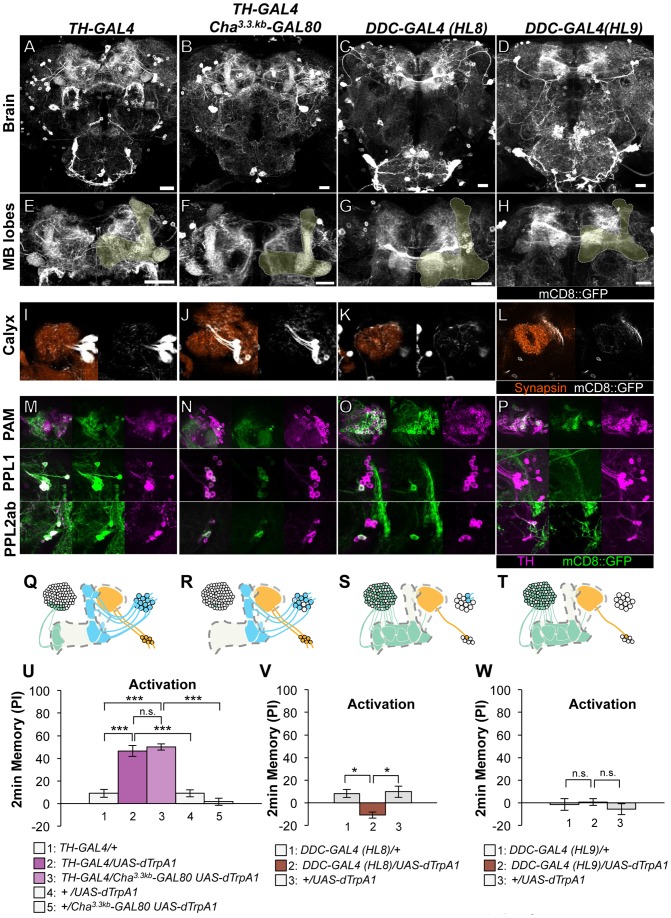
Memories induced by thermo-activation with drivers that label many types of dopamine neurons. (A–D) Expression of mCD8::GFP in the central brain in drivers described above (frontal view; dorsal up). (E–H) Magnification of the anterior brain region including the MB-lobes (shaded). Scale bars represent 20 µm. (I–L) Magnification of the posterior regions centered on the MB calyx. (M–P) TH-immunoreactivity in three clusters of dopamine neurons that project to the MB. (Q–T) Diagrams illustrate the dopamine neurons projecting to the MB labeled in each driver. (U–W) Immediate memories induced by transient thermo-activation of dTRPA1-expressing cells. Memory of experimental group *GAL4/UAS-dTrpA1* is compared with that of control groups (i.e. *GAL4/+, +/UAS-dTrpA1*). For experiments with *GAL80*, the performance of *GAL4/GAL80 UAS-dTrpA1* is compared with those of *GAL4/UAS-dTrpA1*, *GAL4/+* and *+/GAL80 UAS-dTrpA1*. *n* = 9–20. Bars and error bars represent the mean and s.e.m., respectively. * *P*<0.05; *** *P*<0.001; n.s. not significant.

Dopamine biosynthesis requires the enzymes TH and Dopa Decarboxylase (DDC) and these enzymes are expected to be in all dopamine neurons. *TH-GAL4* and two versions of *DDC-GAL4* (*HL8* and *HL9*) label the majority, but not all, dopamine neurons. *TH-GAL4* labels all cells in the PPL1 and PPL2ab clusters and a small subset of the PAM cluster including MB-M3 neurons (Figure 2A, 2E, 2I, 2M, 2Q). Activation of these neurons induced robust aversive memories even in the *dTrpA1* mutant background (Figure 2U, Figure S4), suggesting that activation of dopamine neurons alone can substitute an aversive unconditioned stimulus. *Cha^3.3kb^-GAL80* suppressed transgene expression in some of the PAM cluster cells and three cells in the PPL1 cluster of *TH-GAL4* (Figure 2B, 2F, 2J, 2N, 2R). The silenced cells include MB-M3 neurons and at least one MB-MP1 neuron [25]. *TH-GAL4 Cha^3.3kb^-GAL80/UAS-dTrpA1* flies however showed a similar level of aversive memory as *TH-GAL4/UAS-dTrpA1* flies (Figure 2U). This result implies functional redundancy of *Cha-GAL80*-positive MB-M3 neurons and MB-MP1 neuron in immediate memory.

*HL8* and *HL9* label the majority of PAM cluster cells, one or no PPL1 cells and several PPL2ab cells (Figure 2C–2D, 2G–2H, 2K–2L, 2O–2P, 2S–2T). Although these drivers also label serotonergic neurons, some of which project to the MB [37], [38], terminals in the calyx are TH immunoreactive (Figure S3), suggesting that they belong to the PPL2ab cluster. With *HL8* and *HL9*, dTRPA1 activation did not induce significant aversive memory (Figure 2V–2W), consistent with a previous report using light-dependent activation [19]. Because these drivers label many cell types, the roles of individual dopamine neurons might be obscured in final memory scores by antagonizing functions each other.

**Figure 3:**
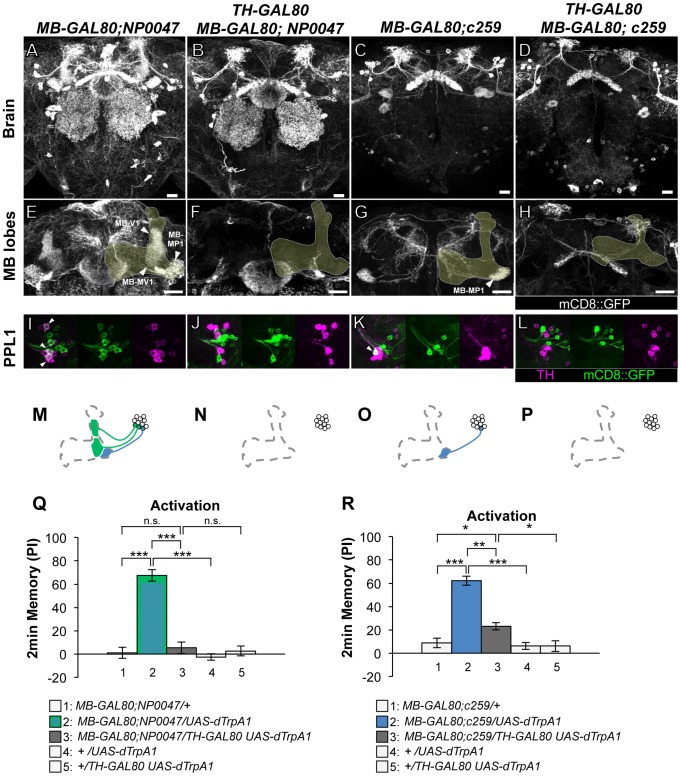
Memories induced by thermo-activation with *MB-GAL80;NP0047* and *MB-GAL80;c259*. (A–D) Expression of mCD8::GFP in the central brain in drivers described above (frontal view; dorsal up). (E–H) Magnification of the anterior brain region including the MB-lobes (shaded). Scale bars represent 20 µm. (I–L) TH-immunoreactivity in the PPL1 cluster of dopamine neurons. Arrowheads indicate colocalization of TH and GFP. (M–P) Diagrams illustrate the dopamine neurons projecting to the MB labeled in each driver. (Q–R) Immediate memories induced by transient thermo-activation of dTrpA1 expressing cells. (Q) *n* = 12–16. (R) *n* = 16–17. Bars and error bars represent the mean and s.e.m., respectively. * *P*<0.05; *** *P*<0.001; n.s. not significant.

As to the PPL1 cluster neurons, two drivers labeling MB-MP1 neuron (i.e. *MB-GAL80;NP0047* and *MB-GAL80;c259*) induced very robust aversive memories (Figure 3), which is fully consistent with the previous result using other drivers *c061;MB-GAL80* and *NP2758* [25]. *TH-GAL80* silenced GAL4 activity selectively in the dopamine neurons in these drivers (Figure 3B, 3F, 3J, 3D, 3H, 3L). Memory induced with these drivers was also significantly suppressed by *TH-GAL80*, although it was not complete with *MB-GAL80;c259* (Figure 3R). Therefore, we used *c061;MB-GAL80*, but not *MB-GAL80;c259*, for the following experiment to manipulate MB-MP1 neuron.

**Figure 4:**
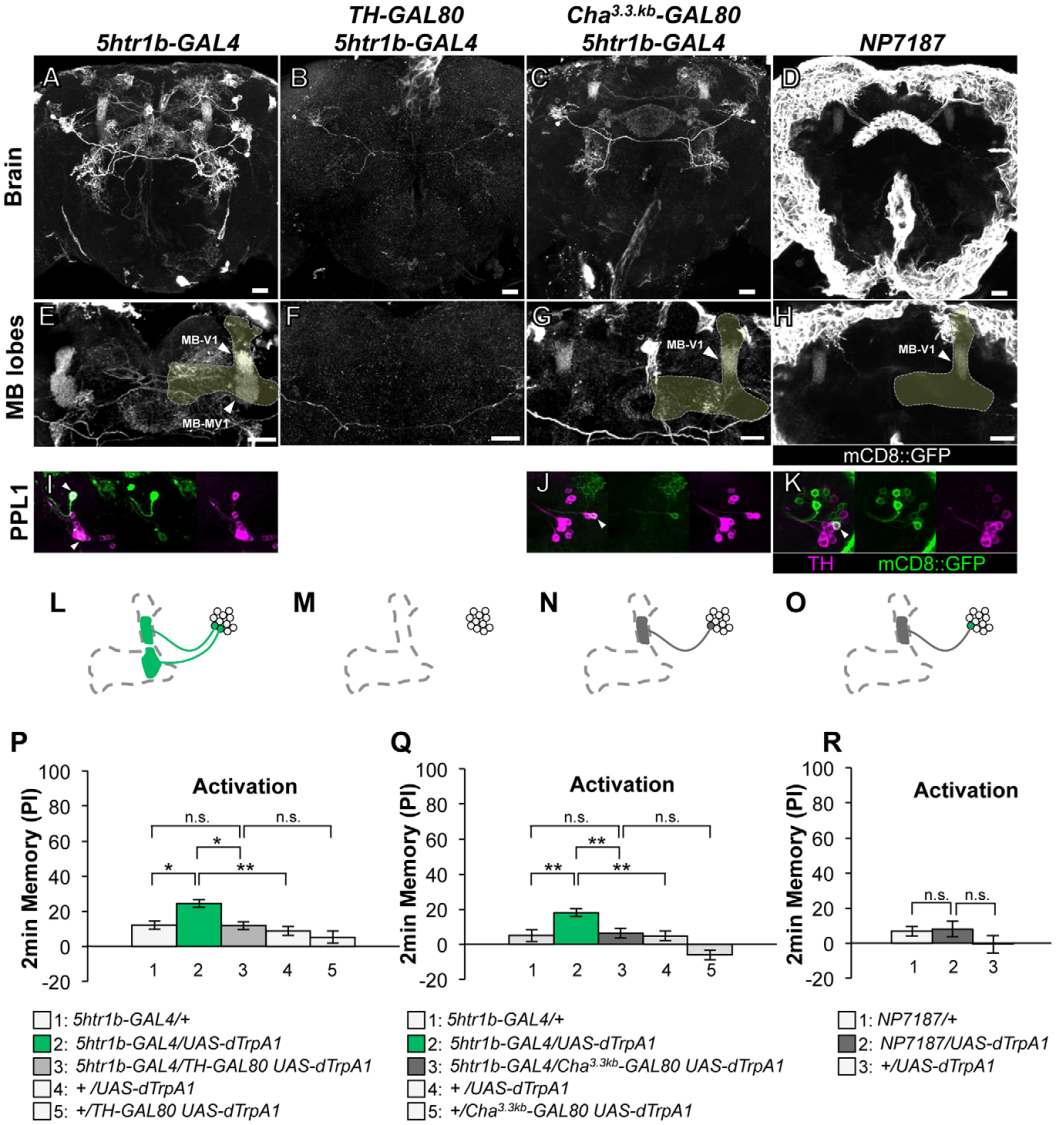
Memories induced by thermo-activation with *5htr1b-GAL4* and *NP7187*. (A–D) Expression of mCD8::GFP in the central brain in drivers described above (frontal view; dorsal up). (E–H) Magnification of the anterior brain regions including the MB-lobes (shaded). Scale bars represent 20 µm. (I–K) TH-immunoreactivity in the PPL1 cluster of dopamine neurons. Arrowheads indicate colocalization of TH and GFP. (L–O) Diagrams illustrate the dopamine neurons projecting to the MB labeled in each driver. (P–R) Immediate memories induced by transient thermo-activation of dTrpA1 expressing cells. (P) *n* = 20–22. (Q) *n* = 16. (R) *n* = 12. Bars and error bars represent the mean and s.e.m., respectively. * *P*<0.05; ** *P*<0.01; n.s. not significant.

The GAL4 driver line *5htr1b-GAL4* labels one MB-V1 neuron and one MB-MV1 neuron in the PPL1 cluster (Figure 4A, 4E, 4I, 4L). *5htr1b-GAL4* induced a slight but significant aversive memory (Figure 4P). *TH-GAL80* suppressed most of the GAL4-positive cells of *5htr1b-GAL4* (Figure 4B, 4F, 4M) and accordingly dTRPA1-induced memory (Figure 4P). We combined *Cha^3.3kb^-GAL80* with *5htr1b-GAL4* that preferentially silenced MB-MV1 neuron but did not abolish expression in MB-V1 neuron and other GAL4-positive cells (Figure 4C, 4G, 4J, 4N). *5htr1b-GAL4 Cha^3.3kb^-GAL80* did not induce memory (Figure 4Q), suggesting the critical role of MB-MV1. Consistently, in *NP7187* and *MZ840*, which label one MB-V1 neuron, activation did not induce significant memory (Figure 4D, 4H, 4K, 4O, 4R) [25]. Thus, memory induced with *5htr1b-GAL4* is likely to be an effect of MB-MV1 neuron activation. In accordance with this interpretation, calcium imaging revealed that MB-MV1 neuron robustly responds to electric shock, whereas MB-V1 neuron preferentially responds to odors [21]. Considering the importance of the vertical lobes in olfactory learning [8], [9], [39], [40], [41], [42], MB-MV1 neuron might act together with MB-V1 neuron during memory acquisition.

**Figure 5:**
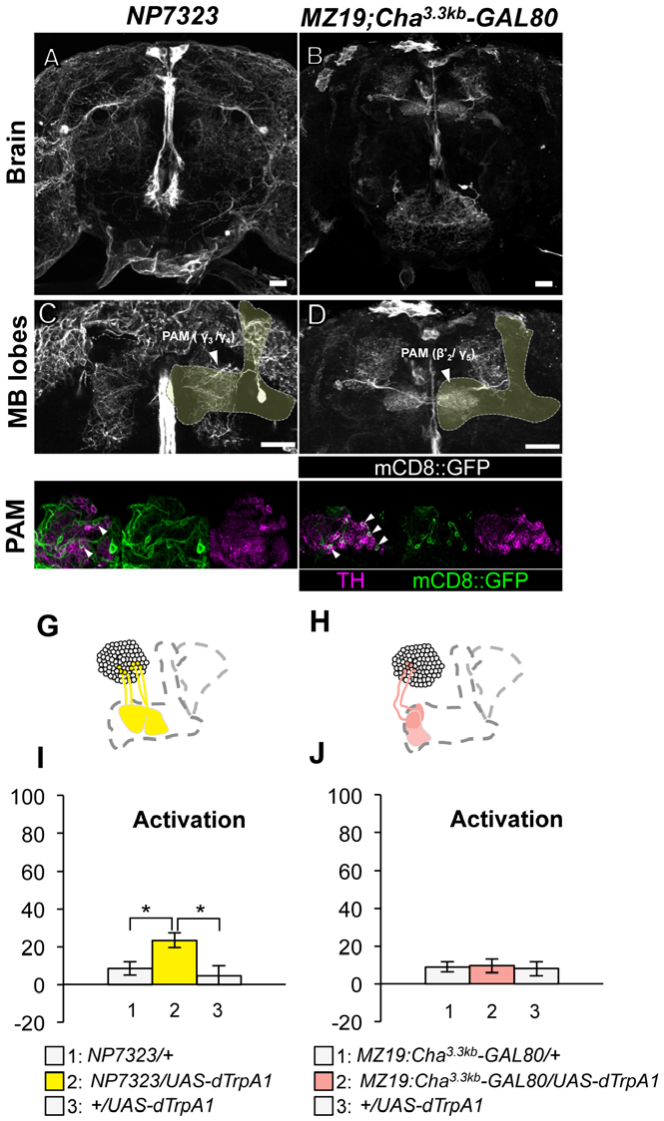
Memories induced by thermo-activation with *NP7323* and *MZ19;Cha^3.3kb^-GAL80*. (A–B) Expression of mCD8::GFP in the central brain in drivers described above (frontal view; dorsal up). (C–D) Magnification of the anterior brain region including the MB-lobes (shaded). Scale bars represent 20 µm. (E–F) TH-immunoreactivity in the PAM cluster of dopamine neurons. Arrowheads indicate colocalization of TH and GFP. (G–H) Diagrams illustrate the dopamine neurons projecting to the MB labeled in each driver. (I–J) Immediate memories induced by transient thermo-activation of dTrpA1 expressing cells. (I) *n* = 14–16. (J) *n* = 12. Bars and error bars represent the mean and s.e.m., respectively. * *P*<0.05; n.s. not significant.

Thermo-activation with *NP7323*, which labels a mixture of cell types in the PAM cluster (MB-M2), induced a slight but significant aversive memory (Figure 5A, 5C, 5E, 5G, 5I). With *MZ19;Cha^3.3kb^-GAL80*, which labels approximately ten PAM cluster cells, activation did not have significant effect (Figure 5B, 5D, 5F, 5H, 5J). These results imply functional heterogeneity in memory formation of PAM cluster neurons. However, we could not test whether the induced memory in *NP7323* can be attributed to non-dopaminergic GAL4-positive neurons, because *TH-GAL80* does not suppress GAL4 expression in the majority of PAM cluster cells.

These results, together with our previous report [25], point to three distinct dopamine pathways to the MB that induce aversive memory; MB-M3 neurons in the PAM cluster and MB-MP1 neuron and MB-MV1/MB-V1 neurons in the PPL1 cluster. The dopamine neurons of the three distinct functional groups terminate in segregated MB subdivisions along the trajectory of Kenyon cell axon bundles: MB-M3 neurons to βsp2 and β'a2, MB-MP1 neuron to γ1 and the core of the peduncle where the α/β neurons innervate, MB-MV1 neuron to γ2 and α'1, MB-V1 neuron to α2, α'2 and a part of α'1 (Figure 1E; see [15] for the description of MB subdivisions). Because blocking all of these neurons with *TH-GAL4* did not abolish shock-induced aversive memory, we reserve the possibility that additional types of neurons may contribute to aversive memory formation (e.g. MB-M2 neurons in *NP7323* and serotonin neurons [35]).

### Anatomical characterization of the dopamine neurons

**Figure 6:**
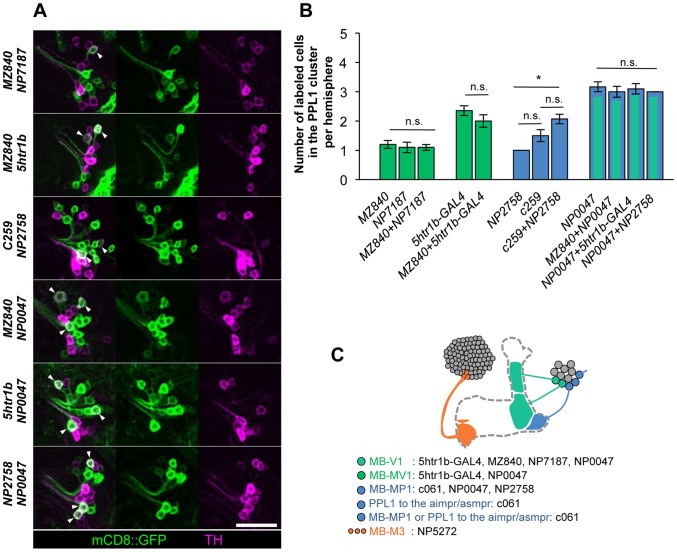
Identity of labeled cells in the PPL1 cluster. (A) TH-immunoreactivity in the PPL1 cluster of dopamine neurons in the combination of GAL4 drivers. Arrowheads indicate colocalization of TH and GFP. Scale bar represents 20 µm. (B) Counting of mCD8::GFP-labeled dopamine neurons in the PPL1 cluster. * *P*<0.05; n.s. not significant. (C) A diagram illustrating the identity and the number of dopamine neurons labeled in respective GAL4 drivers. See [25], [26] for the data of *c061*, *NP2758*, and *MZ840*.

To characterize the individual dopamine pathways to the MB, we determined cellular identity by counting TH-immunoreactive cells in single and combined drivers (Figure 6A–6B). When two drivers label identical dopamine neurons, the number of cells in combined drivers should not differ from those in respective single drivers. Identical MB-V1 neuron is labeled in *5htr1b-GAL4, MZ840, NP7187, NP0047. 5htr1b-GAL4* and *NP0047* label the same MB-MV1 neuron. *c061;MB-GAL80*, *NP0047* and *NP2758* label the same MB-MP1 neuron, while *c061;MB-GAL80* and *MB-GAL80;c259* may label another MB-MP1 neuron in addition to that in *NP2758* and *NP0047* [25].

**Figure 7:**
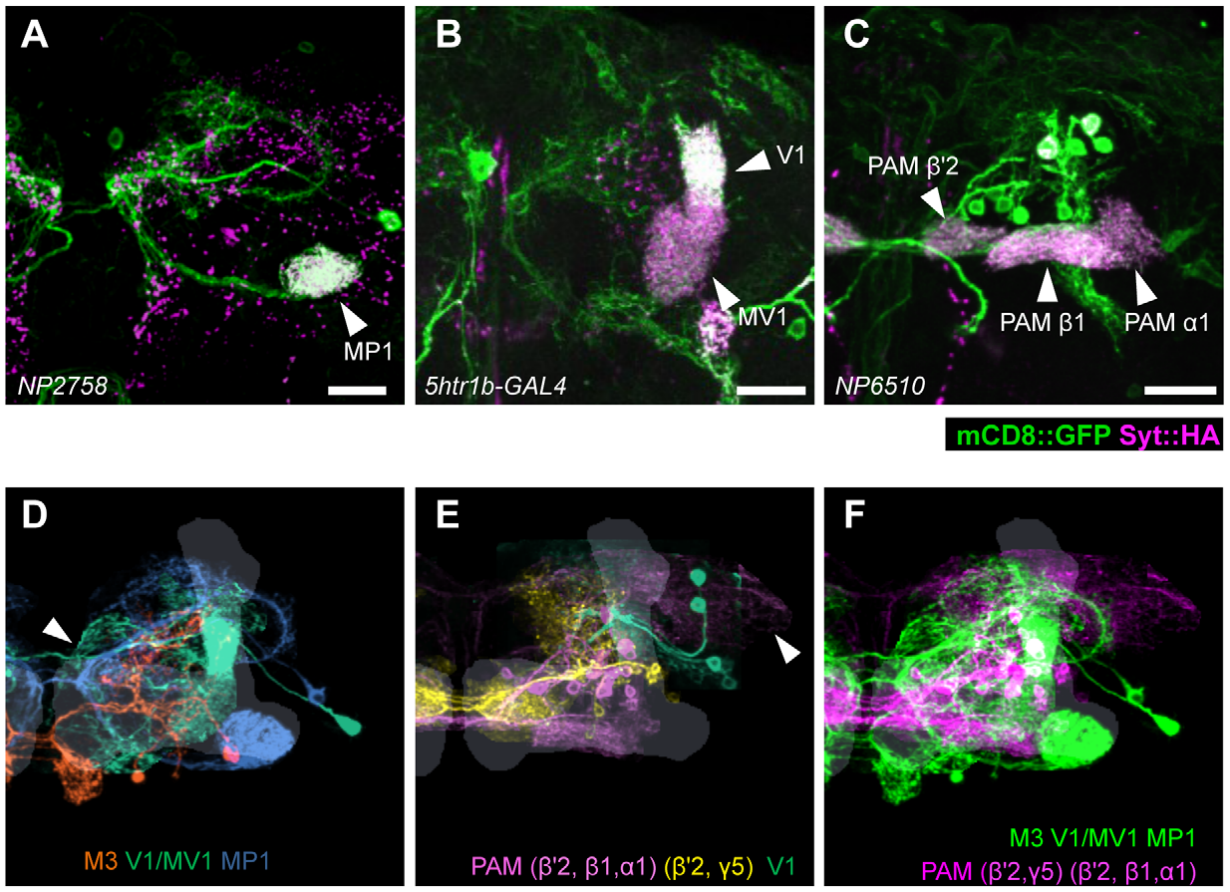
Projection of specific dopamine neurons that induce aversive odor memory. Double labeling of dopamine neurons by membrane and presynaptic markers *UAS-Syt::HA;UAS-mCD8::GFP* driven by (A) *NP2758*, (B) *5htr1b-GAL4*, (C) *NP6510*. Scale bars represent 20 µm. (D) Projection of registered brains labeling dopamine neurons that induce aversive memory. The anterior inferior medial protocerebrum (aimpr) is commonly innervated by these neurons (arrowhead). (E) Registration of control brains that do not induce aversive memory. The prominent processes project in the lateral protocerebrum (arrowhead). (F) The two functional groups of dopamine neurons are separately pooled and presented in different colors (green and magenta, respectively) for comparison. The MB lobes are shown in gray. Scale bars represent 20 µm.

Expression of presynaptic markers in these dopamine neurons revealed that their arbors in the MB are presynaptic terminals (Figure 7A–7C) [25]. The processes of these dopamine neurons in the protocerebrum contained many fewer output sites, implying a dendritic nature (Figure 7A–7C). To compare the distribution of the dendrites, we performed a non-rigid intensity-based transformation of brains [43] and 3D image analysis of the dopamine neurons. We used TH immunolabelling as landmarks to transform brains. Computational alignment of the MB-M3 neurons, MB-MV1/V1 neurons, and MB-MP1 neuron revealed that each cell type has a unique pattern of dendrite distribution in the superior and inferior protocerebral regions, and they are partially overlapping with each other (Figure 7D, Video S1). Furthermore, we aligned the neurons that did not induce significant aversive memory (Figure 4 and Figure 5) and found that their processes outside the MB have different distribution and focus (Figure 7E, Video S2). When the memory-inducing and non-inducing neurons are separately pooled and superimposed in one brain, the dendrites of these groups are largely segregated, especially in the anterior inferior medial protocerebrum (Figure 7F, Video S3). These results imply that the dopamine neurons for aversive memory may partially share some neuronal input that is distinct from dopamine neurons that do not induce aversive memory. Cellular identification of presynaptic neurons requires more precise anatomical and functional analyses.

### Differential requirement of three dopamine pathways for shock-induced memory

**Figure 8:**
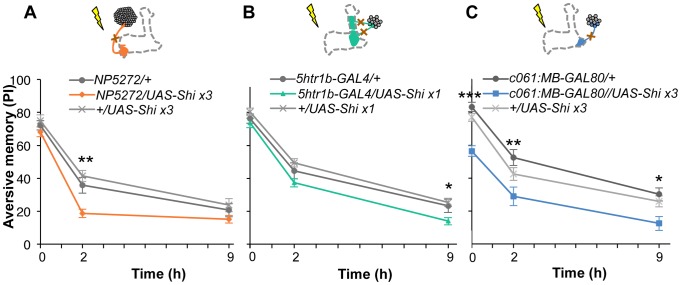
Requirement of the identified dopamine neurons for shock reinforcement. Output of the targeted neurons is blocked with Shi^ts1^ during conditioning by shifting up temperature to 33°C for 30 min prior to the training. Memory is tested immediately following the training or two or nine hours after keeping flies at permissive temperature (25°C). (A) *NP5272/UAS-shi^ts1^* shows significantly impaired 2-hour memory, while the effect on immediate and 9-hour memory is marginal. *n* = 12–16. (B) Blocking with *5htr1b-GAL4* preferentially impaired 9-hour memory, whereas 2-min and 2-hour memory is not significantly affected. Because of memory impairment at permissive temperature with three copies of *UAS-shi^ts1^*, one copy of *UAS-shi^ts1^* was used (see also Figure S5). *n* = 16–28. (C) Memories at all retention times are significantly impaired by blocking with *c061;MB-GAL80*. *n* = 16–24. Bars and error bars represent the mean and s.e.m., respectively. * *P*<0.05; ** *P*<0.01; n.s. not significant.

Based on cell counting and the behavioral screen, we selected drivers *NP5272*, *5htr1b-GAL4*, *c061;MB-GAL80* to further characterize three MB-M3 neurons, one MB-MV1 neuron/one MB-V1 neuron, and one or two MB-MP1 neurons, respectively (Figure 6C). We addressed whether these dopamine neurons function for mediating electric shock reinforcement. To test their necessity during shock conditioning, we transiently blocked corresponding neurons by expressing Shi^ts1^ [44]. Previous studies reported that blocking many dopamine neurons with *TH-GAL4* (including MB-M3, MB-MP1 and MB-MV1/MB-V1 neurons) severely impaired memory irrespective of retention times [19], [25], [45], [46]. Notably, the transient Shi^ts1^ block of each dopamine pathway impaired specific temporal components of aversive memory of shock (Figure 8). The block with *NP5272* preferentially impaired 2-hour memory, but not significantly affected 2-min or 9-hour memory (Figure 8A) [25].

**Figure 9:**
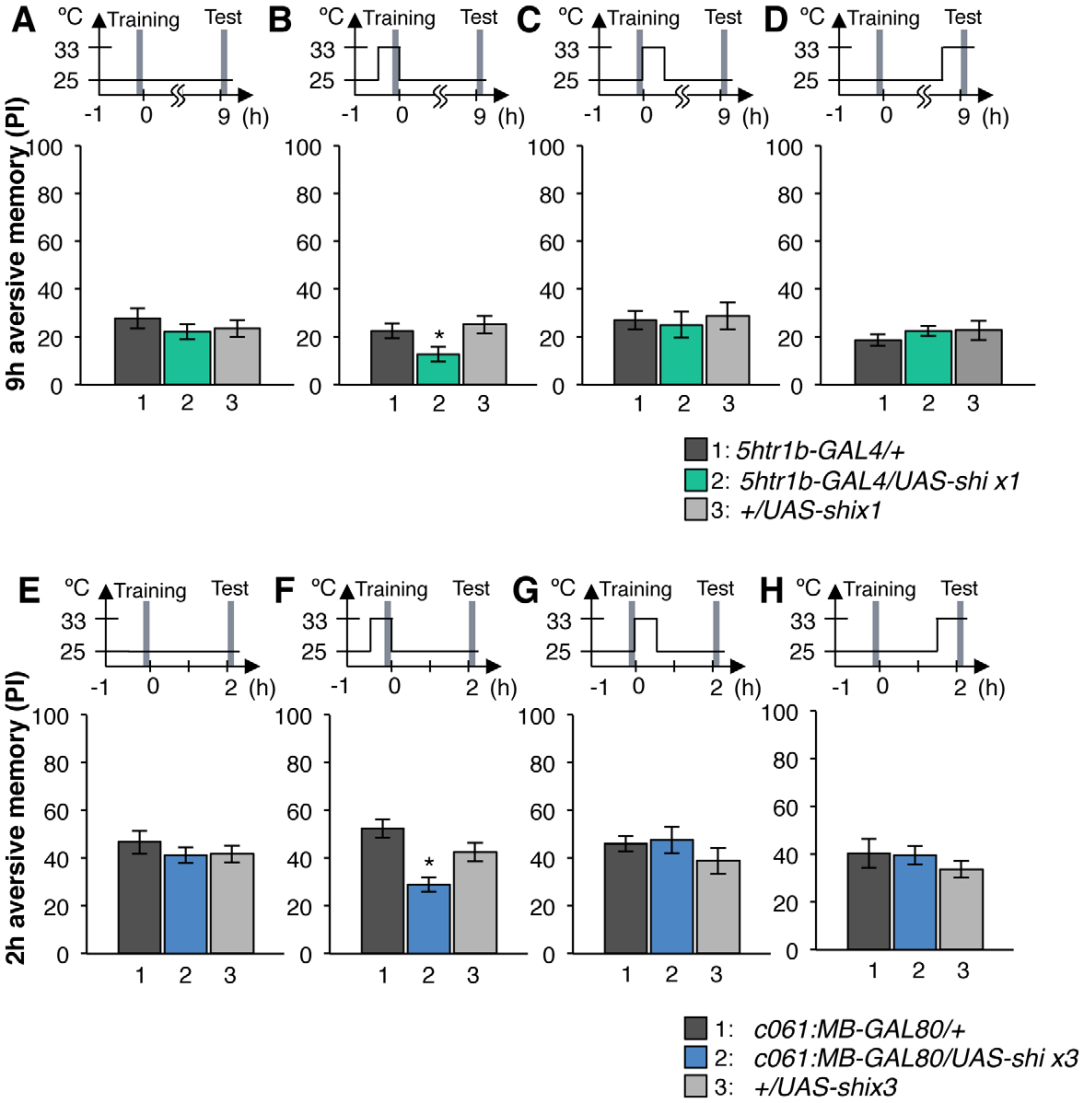
Temporal requirement of the MB-MP1 and MB-MV1/MB-V1 during training. (A–D) Output of MB-MV1/V1 is selectively required for the formation of consolidated memory (9 h). At permissive temperature, *5htr1b-GAL4/UAS-shi^ ts1^* show indistinguishable memory performance compared to the control genotypes (A). The transient block for 30 min including the training phase causes a slight but significant impairment of 9 hour memory (B), while the same blockade immediately after training (C) or during test (D) did not (*n* = 12–28). (E–H) *c061;MB-GAL80/UAS-shi^ ts1^* show impaired 2-h memory, only when they are trained at restrictive temperature (F). Permissive temperature (E), the same temperature shift after training (G), or during test (H) does not significantly affect the memory performance (*n* = 12–20).

We found that the block with *5htr1b-GAL4* with multiple copies of *UAS-shi^ts1^* caused gradual memory impairment over time, leaving the immediate memory intact, although this fly had a significant phenotype at a permissive temperature (Figure S5). We reproduced the impairment of 9-hour memory with a single copy of *UAS-shi^ts1^* (Figure 8B). This impairment of *5htr1b-GAL4/UAS-shi^ts1^* in consolidated memory was due to the transient block during training, since the same inhibition during consolidation or retrieval did not significantly affect the performance (Figure 9A–9D).

In contrast to these defects in selective memory phases, the block with *c061;MB-GAL80* significantly impaired all tested memory phases (i.e. 2 min, 2 hours and 9 hours after training; Figure 8C), although a previous study found no significant impairment of 3-hour memory [26]. This is presumably due to the higher restrictive temperature in this study (33°C compared to 31°C), since the effect of Shi^ts1^ is sensitive to a small temperature difference [47]. The observed memory impairment with *c061;MB-GAL80* should not be attributed to a defect in detecting odor or electric shock itself, as their reflexive avoidance was normal (Figure S6D). Furthermore, blocking after training or experiments at permissive temperature did not cause this phenotype (Figure 9E–9H). We also confirmed that blocking neurons outside our target dopamine neurons by combining GAL80 lines did not impair memory (Figure S6A–S6C and S6E–S6F). Taken together, we propose that electric shock punishment recruits multiple types of dopamine neurons MB-MP1, MB-M3 and MB-MV1/MB-V1 to form parallel memory traces with distinct temporal stability. Additional dopamine neurons might be recruited for signaling aversive reinforcement, since blocking all these dopamine pathways with *TH-GAL4* leaves residual memory in contrast to complete abolishment of memory in dDA1 receptor mutants and neuron-specific *TH* mutants [17], [18], [25], [45], [46].

### Dopamine pathways induce memories with distinct temporal dynamics

**Figure 10:**
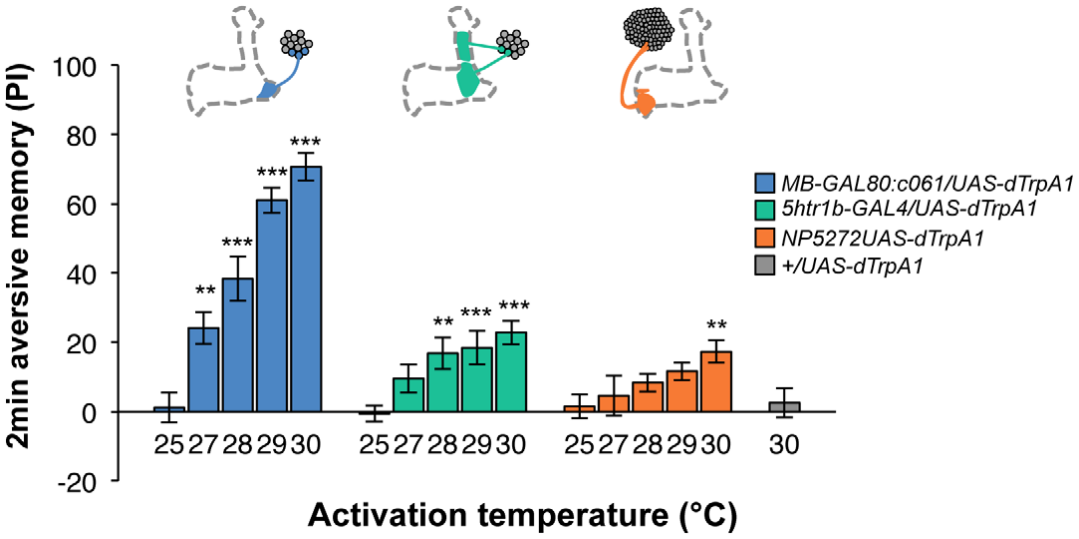
Magnitude of immediate memory depends on the cell type and dTRPA1 activation. Thermo-activation of individual dopamine pathways with various temperatures from 25°C to 30°C induces different degrees of immediate memories. *c061;MB-GAL80/UAS-dTrpA1* formed significant memory at 27°C compared to flies with no activation (25°C). Memory performance steeply increased with the elevation of activation temperature. In contrast, *5htr1b-GAL4* and *NP5272* cause more modest immediate memory with the lowest activation temperature for inducing significant memory at 28°C and 30°C, respectively. Bars and error bars represent the mean and s.e.m., respectively. * *P*<0.05; ** *P*<0.01; *** *P*<0.001 by Dunnett's multiple comparison test with 25°C control following one-way ANOVA. *n* = 12–16.

Contribution of each dopamine neuron to the synthesis of total memory depends on both the magnitude of initial memory and its stability over time. We found that the magnitude of dTRPA1-induced immediate memory depended on the activation temperature and cell type (Figure 10). This implies that the amount of dopamine input represents the strength of reinforcement.

**Figure 11:**
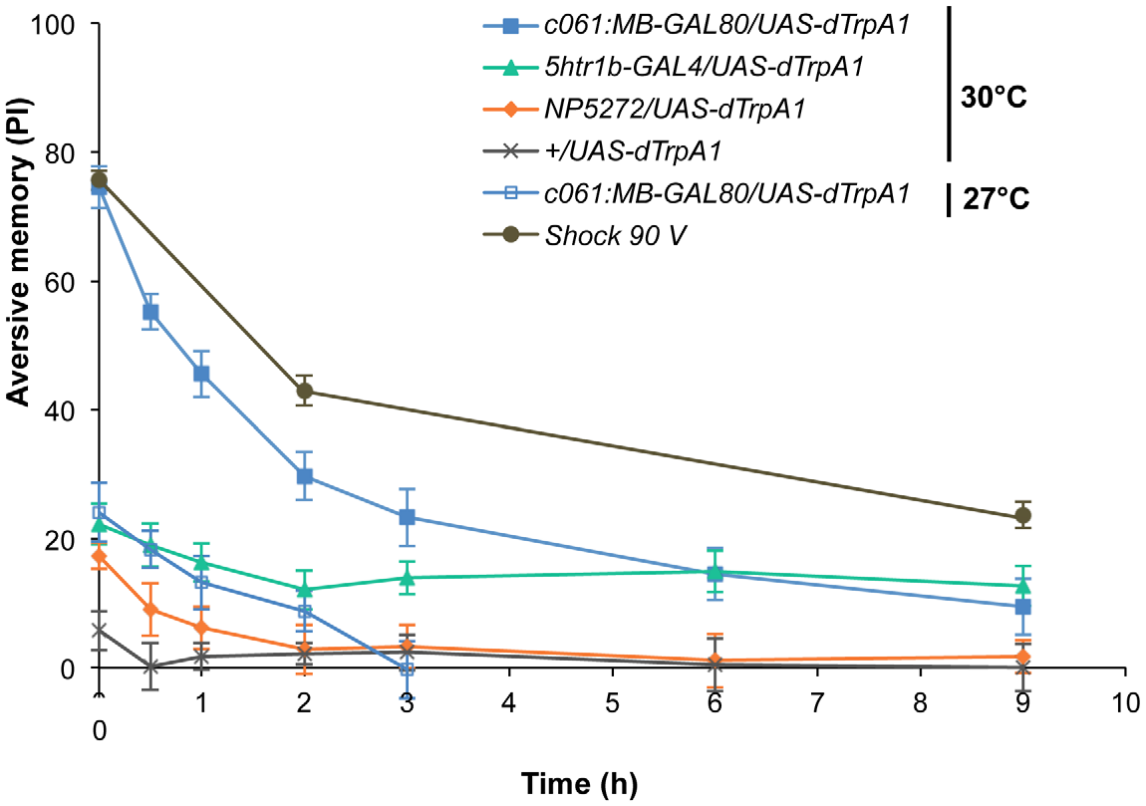
Individual dopamine pathways induce memories with distinct retention dynamics. Flies expressing dTRPA1 in different dopamine neurons are trained at 30°C and tested at various retention times. *c061;MB-GAL80/UAS-dTrpA1* are also trained at 27°C. For comparison, averaged performance of all these genotypes after electric shock conditioning is also plotted (gray; see Figure S7 for memory dynamics of individual genotype). Memories induced by different dopamine neurons decayed with significantly distinct temporal dynamics (*P*<0.05; significant interaction [genotype×retention time] in two-way ANOVA). Points and error bars represent the mean and s.e.m., respectively. *n* = 16–26.

To better understand the role of the distinct reinforcement pathways, we characterized the retention of each memory component in isolation by activating the individual dopamine neurons. Memories induced by these drivers showed remarkable differences in decay dynamics (Figure 11). Initially robust memory induced with *c061;MB-GAL80* decayed rapidly over 9 hours. In contrast, memory induced with *5htr1b-GAL4* was highly stable and still significantly present after 9 hours, although initial memory was moderate. This differential memory decay is not due to the magnitude of initial memory, because the equivalent initial memory with *NP5272* or with milder activation using *c061;MB-GAL80* disappeared completely within 3 hours. In contrast to the similar decay dynamics of memories induced with *NP5272* and *c061;MB-GAL80* (Figure 11), the requirement of these neurons for the retention of shock-induced memories is qualitatively different (Figure 8). The genetic background or the effect of dTRPA1 at permissive temperature is an unlikely cause of the faster/slower memory decay, because the memory retention of the dTRPA1-expressing flies was indistinguishable when trained with electric shock (Figure S7). Thus, these results suggest that each dopamine pathway to the MB establishes a memory component with unique temporal stability.

### Functional synergy and redundancy of multiple memories

The results of activation and inactivation experiments appear to be inconsistent: A simple sum of memory performances by activation of these pathways does not explain selective memory impairments when they are blocked (Figure 8 and Figure 11). For instance, the effect of the blocking MB-M3 neurons with *NP5272* was most pronounced for 2-hour memory (Figure 8A), whereas memory induced by activation of the same cells with *NP5272* did not last 2 hours (Figure 11). The reason of this apparent discrepancy could be technical, such as the copy number of the effectors and temperature regimes. Alternatively it may suggest a synergistic interaction between memories induced by MB-M3 neurons and other dopamine neurons. We noticed another case of possible interaction that suggests functional redundancy; activation with *NP5272* or *5htr1b-GAL4* induced immediate memory (Figure 11), whereas the block with these drivers did not show significant short-term defect (Figure 8A–8B).

**Figure 12:**
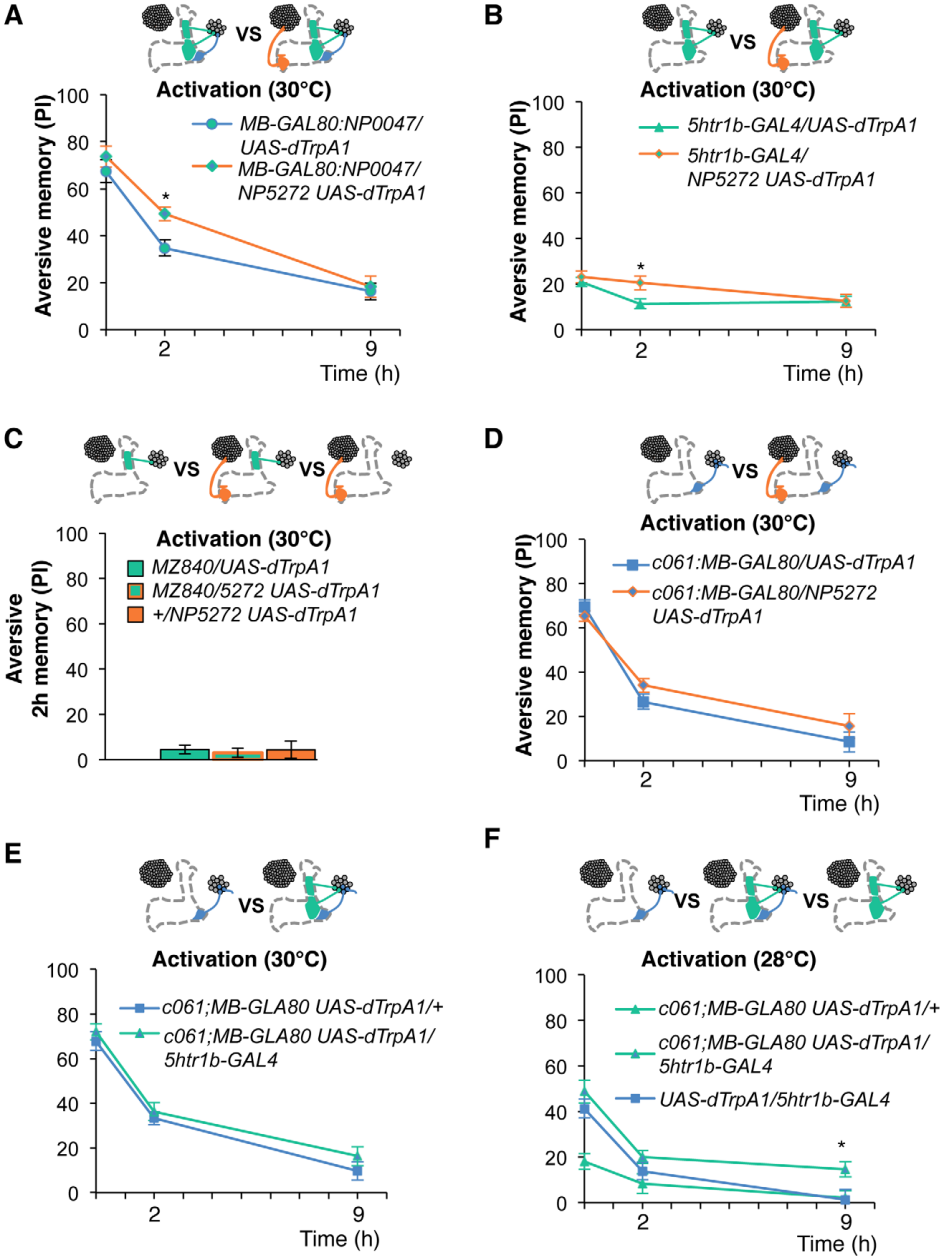
Combinatorial activation of different dopamine neurons shapes the stability of odor memory. Retention of memories by activating different combinations of dopamine neurons. Diagram above each graph depicts dopamine neurons targeted by the driver combination. (A) Memory of *MB-GAL80;NP0047/NP5272 UAS-dTrpA1* is significantly higher than that of *MB-GAL80;NP0047/UAS-dTrpA1* at 2 hours, but not at the other tested time points. *n* = 16–22. (B) Memory of *5htr1b-GAL4 NP5272/UAS-dTrpA1* is significantly higher than that of *5htr1b-GAL4/UAS-dTrpA1* specifically at 2-hour retention. *n* = 16–26. (C) Flies do not form significant 2 h memory with *MZ840* that label MB-V1, but not MB-MV1. The addition of *NP5272* does not improve the memory. *n* = 16. (D) Memory of *c061;MB-GAL80 NP5272/UAS-dTrpA1* is not significantly different from that of *c061;MB-GAL80/UAS-dTrpA1* at all retention time, although it tended to be higher. *n* = 16–26. (E) Compared to *c061;MB-GAL80/UAS-dTrpA1*, combinatorial activation with *c061;MB-GAL80 5htr1b-GAL4* does not significantly improve the performance at all retention times, if cells are activated at 30°C. *n* = 16. (F) Milder dTRPA1-activation at 28°C reveals that the performance of *c061;MB-GAL80/UAS-dTrpA1* and *5htr1b-GAL4/UAS-dTrpA1* is redundant and interdependent for immediate and 9-hour memories, respectively. This resembles the increasing memory impairments upon blocking with *5htr1b-GAL4* (Figure 8B). *n* = 16–22. Bars and error bars represent the mean and s.e.m., respectively. * *P*<0.05; n.s. not significant.

**Figure 13:**
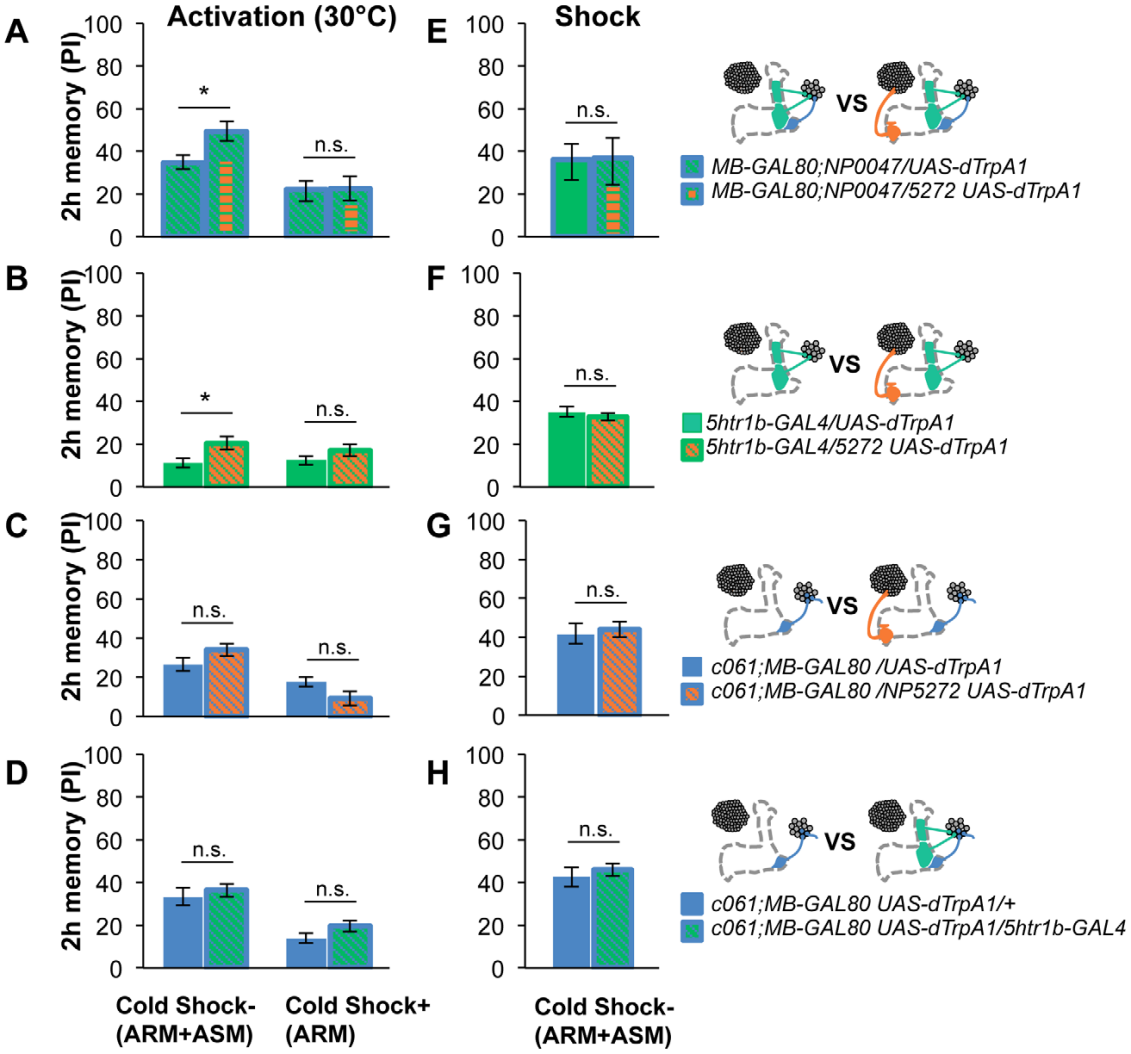
ARM after combinatorial activation of different dopamine neurons. (A–D) Flies are trained by transient activation of dTrpA1 expressing cells using respective GAL4 drivers and tested 2 hours later. (A) *n* = 12–26. (B) *n* = 26. (C) *n* = 16–26. (D) 16–24. (E–H) At permissive temperature (25°C), 2-h memory with electric shock is not significantly different in flies expressing *dTrpA1* with single and specific combinations of drivers that are used for thermo-activation. Bars and error bars represent the mean and s.e.m., respectively (*n* = 12–18). * *P*<0.05; n.s. not significant. For (E), bars and error bars represent median and interquartile range, because the data points were not normally distributed.

To test the various forms of interaction between memory components, we first measured activation of MB-M3 neurons together with other reinforcement pathways. To activate MB-MP1, MB-MV1 and MB-V1 neurons together, we took another driver *MB-GAL80;NP0047* from the activation screening (Figure 3Q), and measured the retention of dTRPA1-induced memory. In comparison to *MB-GAL80;NP0047* alone, additional activation with *NP5272* did not significantly improve the performance of immediate memory (Figure 12A), supporting the redundancy of MB-M3 neurons and the others for immediate memory. This is in line with no significant requirement of MB-M3 neurons for immediate memory induced by electric shock (Figure 8A). In contrast, combinatorial activation significantly improved performance in 2-hour retention (Figure 12A), suggesting the synergistic contribution of MB-M3 neurons to 2-hour memory and recapitulating the selective impairment of 2-hour shock memory upon blocking with *NP5272* (Figure 8A). Also combinatorial activation of MB-M3 and MB-V1/MB-MV1 neurons with *NP5272* and *5htr1b-GAL4* caused a similar pattern of interaction in immediate and 2-hour memories (Figure 12B). In contrast, activation of MB-M3 and MB-V1 neurons without MB-MV1 neuron in *NP5272* and *MZ840* did not induce any 2-hour memory (Figure 12C). Also *NP5272* activation was not significantly additive across all tested retention times compared to *c061;MB-GAL80* single activation (Figure 12D). Thus, we propose that MB-MV1, but not MB-MP1 or MB-V1 neurons, likely interacts with MB-M3 neurons for 2-hour memory. The enhancement of 2-hour memory was likely due to an increase of anesthesia-sensitive memory (ASM), because the effect disappeared by 9 hour when the memory is primarily consisted of anesthesia resistant memory (ARM). Indeed, MB-M3 neurons activation did not contribute to the increase of ARM (Figure 13A, 13C). Memories induced by thermo-activation with *5htr1b-GAL4* and *c061;MB-GAL80* were at least partially ARM (Figure 13).

Combinatorial thermo-activation with *5htr1b-GAL4* and *c061;MB-GAL80* was not significantly different from single activation by *c061;MB-GAL80* (Figure 12E). Milder activation at 28°C however revealed interaction (Figure 12F) that features gradual memory impairments upon blocking with *5htr1b-GAL4* (Figure 8B). Ectopic expression of dTRPA1 or its effect at permissive temperature alone is an unlikely cause of the interaction, since 2-hour memory of all these genotypes was normal when they were trained with electric shock (Figure 13E, 13F, 13G, 13H). Altogether, these results suggest that MB-MV1 and MB-M3 neurons are redundant for immediate memory when MB-MP1 neuron is activated, and different modulatory interactions of MB-MV1/MB-M3 neurons and MB-MV1/MB-MP1 neurons tune the stability of memory.

## Discussion

### Different memory traces in the MB via distinct dopamine neurons

Using freely behaving animals, we demonstrated that at least three essential dopamine pathways to the MB can together synthesize aversive memory that shares similar temporal characteristics with shock-induced memory. They arborize in the different subdomains in the MB. Functional imaging of dopamine neurons revealed that response to electric shock significantly differ between cell types [21]. These results indicate shock reinforcement recruits a specific set of the dopamine pathways to the MBs and induces multiple memory traces in Kenyon cells through different receptors [18], [30], [32]. The *Drosophila* MB is subdivided into domains, that are defined by specific combinations of intrinsic and extrinsic neurons (Figure 1A) [15]. Each dopamine pathway for memory induction intersects the specific axonal compartment of Kenyon cells (Figure 1E, Figure 7A–7B) [15], [21], and we found that the memory components induced by the distinct dopamine neurons interact to tune the stability of collective memory (Figure 12). Therefore, multiple memory traces formed in spatially segregated synapses in the MB would interact with each other.

One of the possible underlying mechanisms is intracellular interaction of the dopamine inputs to a single Kenyon cell. In the sensory neurons of *Aplysia*, simultaneous application of serotonin to different parts of a neuron - the cell body and presynaptic terminals of a sensory neuron - synergistically induces intermediate and long-term facilitation of the sensorimotor synapses [48]. Alternatively, subcellular memory traces may interact at the circuit level via interneurons, such as the DPM and APL neurons, for which previous studies showed their essential role in memory consolidation [6], [38], [49], [50]. A recent behavioral and imaging study revealed that the spontaneous activity of MB-MV1 and MB-MP1 after olfactory conditioning is coordinated to control the stability of memory [51]. Such network level interaction can also be implemented as a neuronal integration of outputs from the multiple memories. Considering parallel formation and the interaction of distinct memory components by different Kenyon cell populations [7], [30], [31], [32], [33], [52], the latter scenario might be more likely.

### Recruitment of dopamine neurons by noxious stimuli

A combined neuroanatomical and computational analysis identified that the dendrites of MB-M3, MB-MV1 and MB-MP1 neurons form a cloud cluster in the protocerebrum, and they project into the segregated domains of the MB (Figure 7). This neuronal configuration implies that a punishment signal undergoes parallel processing in the MB via distinct dopamine pathways to induce different memory traces. The response profile of MB-projecting dopamine neurons is indeed distinct, suggesting that they receive input from different neurons [21]. Characterization of presynaptic neurons innervating the dendritic regions of the dopamine neurons will help identifying a cellular mechanism of parallel processing of reinforcement.

### Multiple roles of dopamine neurons

Recent studies including our results highlight distinct functions of single dopamine neurons. Especially, MB-MP1 neurons are required for: 1) Suppression of the retrieval of appetitive memory when flies are not starved [26]; 2) Formation of aversive odor memory by mediating electric shock (this study); 3) Regulation of long-term memory by synchronized spontaneous activity together with MB-MV1 after spaced training [51]. Although these functions seem incompatible at the first glance, we suggest context-dependent roles of single dopamine neurons.

Functions of MB-MP1 neurons in suppression of conditioned odor approach and aversive reinforcement signaling may be reasonable, as it is not appropriate timing for an animal to follow appetitive memory when challenged by a noxious stimulus (aversive reinforcement). Implementing these two functions to the same MB-extrinsic neuron can be an elegant design by evolution, since both appetitive and aversive memories are formed and stored in the MB. Furthermore, in the light of a recent study, the results of MB-MP1 neurons suggests that dopamine may play opposing roles in the formation and the degradation of ARM (Figure 13) [51]. A key difference of the opposing actions of PPL1 neurons is that Kenyon cells are simultaneously activated by an odor. Therefore, the effect of dopamine release from MB-MP1/MB-MV1 neurons on Kenyon cell synapses might be dependent on the state of the cells. These distinct modes of dopamine action may be better characterized by physiological means, such as functional imaging or electrophysiology.

## Materials and Methods

### Fly strains

We generated flies for behavioral and anatomical studies using the GAL4/GAL80 lines and transgenes listed in Table S1. *5htr1b-GAL4* (II) was generated with the enhancer fragment of *5htr1b* gene. The fragment (−542–+2606) was amplified using the primer pair GTC AAATTCGGTCTGGCATT and CTTGCCTATGATGGTGACG by PCR and cloned into the pCRII-TOPO® vector (Invitrogen). After verifying the sequence, the fragment was cloned into the p221-4 GAL4 vector (gift from E. Knust). *UAS-UAS-Shi^ts1^* ×3 is a combination of P-element insertion on X and multiple insertions on third chromosome, which is identical to Shi2 in [44], [47]. UAS-*Shi^ts1^* ×1 is isolated from multiple insertions on third chromosome by recombination, obtained from T. Préat lab. For experiments with *UAS-Shi^ts1^* and *UAS-dTrpA1*, flies were raised at 18°C and 25°C, respectively, at 60% relative humidity. *UAS-Shi^ts1^* and *UAS-dTrpA1* flies were aged 8–14 and 7–12 days after eclosion, respectively, to allow sufficient accumulation of effecter proteins without age-related memory impairment. For anatomical studies, females of 5–10 days after eclosion at 25°C were analyzed.

### Behavioral assays

For olfactory conditioning, we used 4-methylcyclohexanol and 3-octanol diluted in the paraffin oil (1∶10). One odor was presented for 1 min at elevated temperature or with 12 pulses of electric shocks (90 V) (Figure 1D). Subsequent to 1-min air flush, another odor was presented for 1 min. The reciprocal group of flies was trained by the protocol in which the identity of odors was altered. After a given retention time, the conditioned odor response was measured in a T-maze for two minutes. Then, a performance index (PI) was calculated by taking the mean preference of the two reciprocal groups [53]. The first odor was paired with reinforcement in a half of experiments and the second odor was paired with reinforcement in another half so that the effect of the order of reinforcement is canceled [53]. The protocol for conditioning with thermo-activation by dTRPA1 was essentially same as the standard protocol of olfactory conditioning using electric shock [45], [53], [54], except that flies were transferred to the pre-warmed T-maze in the climate box only during the presentation of one of the two odorants (60 sec) [25]. To minimize the noxious effect of heat itself, we used moderate temperature (30°C) for activation. This temperature shift by itself scarcely induced a significant memory in control genotypes [25]. For measuring the ARM, trained flies were transferred into pre-cooled tube on ice for 60 sec at 100 min after training, then transferred back to the pre-warmed tube at 25°C.

### Statistics

Statistical analyses were performed using Prism (GraphPad Software). Most of the tested groups did not violate the assumption of the normal distribution and the homogeneity of variance. Therefore, mean performance indices were compared with *t*-test, or Dunnett's multiple comparison test or Bonferroni-post test for selected pairs following one-way or two-way ANOVA. For the groups that violated the assumption of parametric statistics, Mann-Whitney test (Figure 13E) or Dunn's multiple comparison test following Kruskal-Wallis-test was applied (Figure 9D).

### Immunohistochemistry

The brain and thoracicoabdominal ganglion were prepared for immunolabeling and analyzed as previously described [16], [25], [54]. For dopamine staining (Figure S1), brains were fixed with 0.6% glutaraldehyde in PBS for 30 min; the unreacted aldehyde groups were subsequently reduced with 0.1% (w/v) sodium tetraborhydrate. Brains were embedded in 7% agarose and thick (150 um) sections were obtained with Laica Vibratome and labelled with the polyclonal antibody against conjugated dopamine (MoBiTech). Frontal optical sections of brain samples were taken with confocal microscopy, Olympus FV1000 or Leica SP2. For evaluating the effect of GAL80, brains to be compared were scanned with identical microscopy setting. Images of the confocal stacks were analyzed with the open-source software Image-J [55].

### Image registration

Intensity-based affine and non-rigid registration of whole brains were performed with the toolbox elastix [43]. Confocal images of entire brains of GAL4/UAS-mCD8::GFP were acquired at 1024×512 pixel resolution. All brains were registered to a representative brain using counterstaining with TH as a reference channel. TH signals are mainly composed of sparse bright landmarks and a detectable background, allowing affine registration. The transformations computed with the TH channel were then applied to the mCD8::GFP channel. For some samples, non-rigid registration based on BSpline interpolation has been applied following affine transformation. Critical parameters, such as grid spacing, were empirically optimized. The accuracy of the registration was evaluated manually based on the matching of anti-TH signals. To compare positions of dendritic arbors of dopamine neurons, we selected samples with a small registration error (<20 µm) and signal from the other GAL4 expressing cells was manually masked (see Figure 4A, Figure 5B, and [25] for the GAL4 expression patterns), and mCD8::GFP channels of different drivers were blended as different colors using ImageJ. The final images in Figure 7D–7F and Videos S1, S2, S3 show only regions surrounding the MB. Employed GAL4 drivers: *NP5272, NP2758, 5htr1b-GAL4* (Figure 7D and Video S1); *MZ840, NP6510* and *MZ19;Cha^3.3kb^-GAL4* (Figure 7E and Video S2); *NP5272*, *NP2758*, *5htr1b-GAL4*, *NP6510* and *MZ19;Cha^3.3kb^-GAL4* (Figure 7F and Video S3).

## Supporting Information

Figure S1 Dopamine immunoreactivity in the brain and PPL1 cluster. The frontal projection view of the posterior half of the brain. Similar to TH-imunoreactivity (Figure 2M), neurons expressing mCD8::GFP in *TH-GAL4* are colabeled by dopamine itself. Scale bar represents 20 µm.
(PDF)

Figure S2 A fraction of GAL4-positive cells in each dopamine cell cluster. (A) A gray scale plots a percentage of GAL4 expressing cells in each cluster. The number in the bracket associated with each cluster denotes the total number of TH-immunoreactive cells per hemisphere (the total number in the brain for unpaired clusters: VUM1 and VUM2). Drivers that induced significant aversive memory are underlined (Figure 2, Figure 3, Figure 4, Figure 5, and [25]).
(PDF)

Figure S3 *DDC-GAL4* (*HL8*) labels TH-immunoreactive processes in the calyx. Confocal stack of the calyx region showing TH-positive processes of *DDC-GAL4* (*HL8*), presumably originating from the PPL2ab cluster. See also Figure 2K–2L. Part of these processes in the calyx have been reported as serotonergic neurons [38]. Scale bar represents 20 µm.
(PDF)

Figure S4 Thermo-activation of dopamine neurons without endogenous *dTrpA1*. Flies were trained by transient activation of dTrpA1 expressing cells as in Figure 1D. Irrespective of the lack of the endogeneous *dTrpA1*, immediate memory of *TH-GAL4/UAS-dTrpA1* is significantly higher than that of control genotypes (*TH-GAL4/+* and *+/UAS-dTrpA1*). Immediate memory of *TH-GAL4/UAS-dTrpA1* in dTrpA1 mutant background (*dTrpA1^ins^*) does not differ from that in wild type background, suggesting that activation of endogeneous dTrpA1 has only negligible effect on memory formation under this experimental condition (60 s elevation to 30°C). *n* = 10–12.
(PDF)

Figure S5 Multiple copies of *UAS-shi^ts1^* preferentially impairs the acquisition of 2-hour memory in *5htr1b-GAL4*. (A) Immediate memory of *5htr1b-GAL4/UAS-shix3* (multiple copies of *UAS-shi^ts1^*) is indistinguishable from the control groups. *n* = 14–16. (B) 2-h memory of *5htr1b-GAL4/UAS-shix3* is significantly affected. *n* = 16. (C) *5htr1b-GAL4/UAS-shix3* also show memory impairment at permissive temperature presumably due to the leaky effect of Shi^ts1^ at high expression level (see also Figure 9A). *n* = 12.
(PDF)

Figure S6 Requirement of MB-MV1/V1 and MB-MP1. (A) With *Cha^1.2kb^-GAL80* that silence expression in both MB-MV1 and MB-V1, *5htr1b-GAL4/UAS-shi^ts1^*does not show significant impairment of 9-hour memory (*n* = 20–28). (B–C) mCD8::GFP signals in the central brains with respective drivers (frontal view; dorsal up). *Cha^1.2kb^-GAL80* silences expression in the PPL1 cluster cells in *5htr1b-GAL4* and *c061*. Scale bars represent 20 µm. (D) Blocking MB-MP1 neurons with *c061;MB-GAL80* does not impair avoidance of electric shock, MCH or OCT (*n* = 12). (E) *TH-GAL80* or *Cha^1.2kb^-GAL80*, *c061;MB-GAL80/UAS- shi^ts1^*does not show a significant memory impairment at 2 hour retention (*n* = 20–26). (D) The block with *c061;MB-GAL80* driving a single copy of *UAS- shi^ts1^* transgene results in a similar impairment of 2 hour memory, which is restored with *Cha^3.3kb^-GAL80* (*n* = 12). See [25] for the expression pattern of *c061;MB-GAL80* in combination with *Cha^3.3kb^-GAL80*. Bars and error bars represent the mean and s.e.m., respectively. * *P*<0.05; ** *P*<0.01; n.s. not significant.
(PDF)

Figure S7 No significant effect of *dTrpA1* expression in dopamine neurons at permissive temperature. At permissive temperature (25°C), flies expressing *dTrpA1* with *NP5272*, *5htr1b-GAL4*, *c061;MB-GAL80* or no driver show indistinguishable levels of shock-induced memories at all the tested time points. *n* = 10–14.
(PDF)

Table S1 List of crosses for behavior experiments.
(DOC)

Video S1 Dopamine neurons that induce aversive memory. 3-D rotation movie of the data shown in Figure 7D.
(AVI)

Video S2 Dopamine neurons that do not induce aversive memory. 3-D rotation movie of the data shown in Figure 7E.
(AVI)

Video S3 Comparison of dopamine neurons that induce aversive memory and those do not. 3-D rotation movie of the data shown in Figure 7F.
(AVI)
