# Supplementary figures and images for: Three Dopamine Pathways Induce Aversive Odor Memories with Different Stability
Source: PLoS Genet. 2012 Jul 12;8(7):e1002768. doi: 10.1371/journal.pgen.1002768 (PMC3395599; doi:10.1371/journal.pgen.1002768)

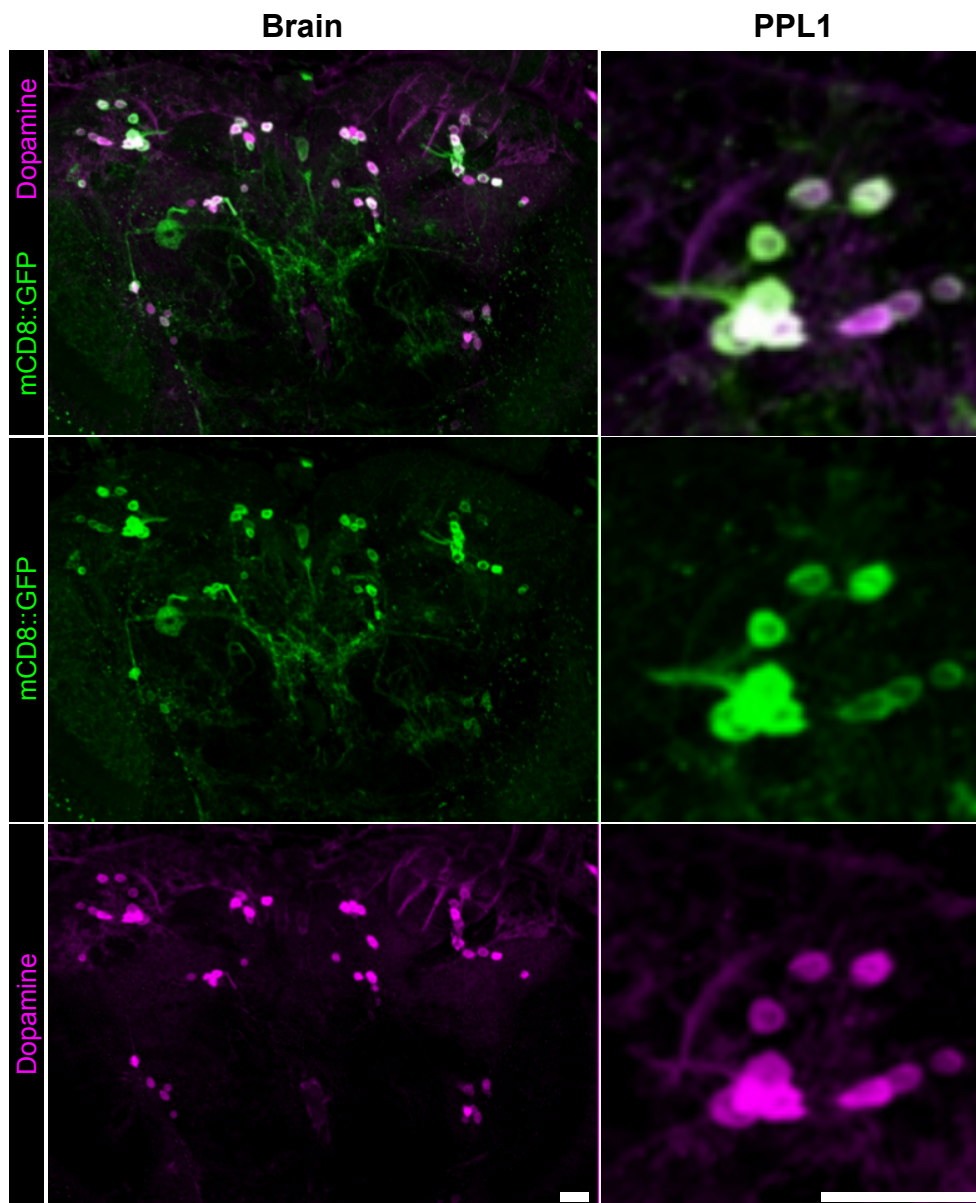

Aso et al., Figure S1

Supplement: Figure S1 — Dopamine immunoreactivity in the brain and PPL1 cluster. The frontal projection view of the posterior half of the brain. Similar to TH-imunoreactivity (Figure 2M), neurons expressing mCD8::GFP in TH-GAL4 are colabeled by dopamine itself. Scale bar represents 20 µm. (PDF) [file pgen.1002768.s001.pdf]

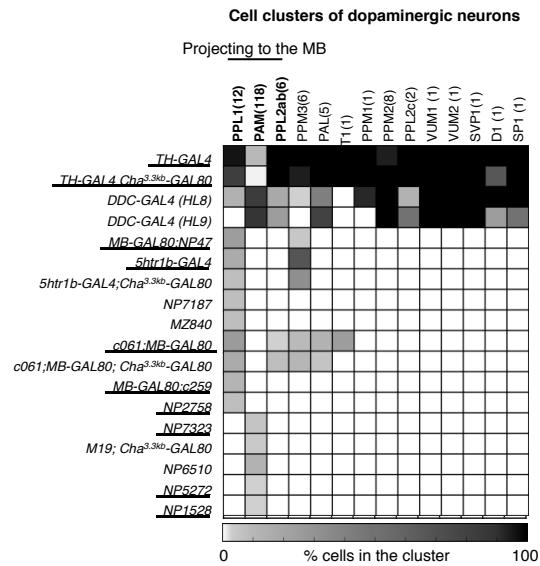

**Aso et al., Figure S2**

Supplement: Figure S2 — A fraction of GAL4-positive cells in each dopamine cell cluster. (A) A gray scale plots a percentage of GAL4 expressing cells in each cluster. The number in the bracket associated with each cluster denotes the total number of TH-immunoreactive cells per hemisphere (the total number in the brain for unpaired clusters: VUM1 and VUM2). Drivers that induced significant aversive memory are underlined (Figure 2, Figure 3, Figure 4, Figure 5, and [25]). (PDF) [file pgen.1002768.s002.pdf]

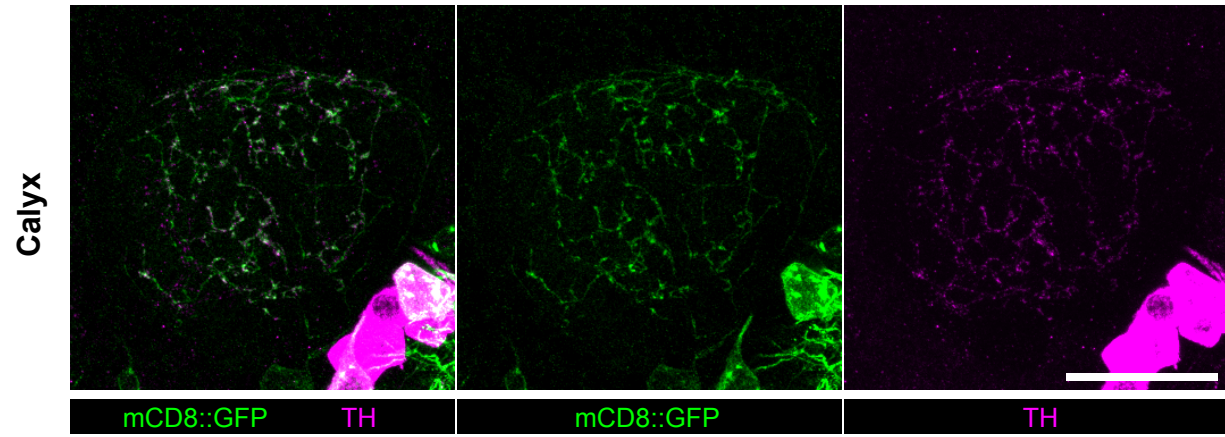

Aso et al., Figure S3

Supplement: Figure S3 — DDC-GAL4 (HL8) labels TH-immunoreactive processes in the calyx. Confocal stack of the calyx region showing TH-positive processes of DDC-GAL4 (HL8), presumably originating from the PPL2ab cluster. See also Figure 2K–2L. Part of these processes in the calyx have been reported as serotonergic neurons [38]. Scale bar represents 20 µm. (PDF) [file pgen.1002768.s003.pdf]

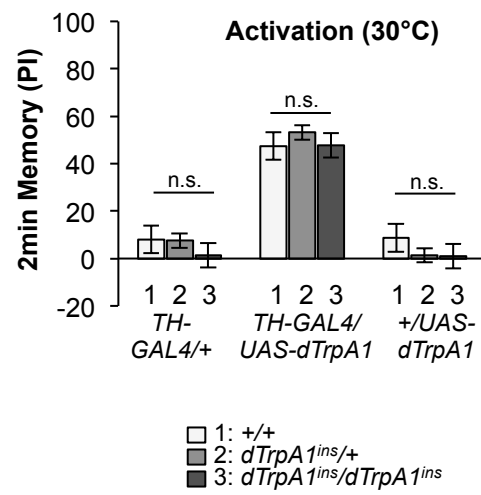

**Aso et al., Figure S4**

Supplement: Figure S4 — Thermo-activation of dopamine neurons without endogenous dTrpA1. Flies were trained by transient activation of dTrpA1 expressing cells as in Figure 1D. Irrespective of the lack of the endogeneous dTrpA1, immediate memory of TH-GAL4/UAS-dTrpA1 is significantly higher than that of control genotypes (TH-GAL4/+ and +/UAS-dTrpA1). Immediate memory of TH-GAL4/UAS-dTrpA1 in dTrpA1 mutant background (dTrpA1ins) does not differ from that in wild type background, suggesting that activation of endogeneous dTrpA1 has only negligible effect on memory formation under this experimental condition (60 s elevation to 30°C). n = 10–12. (PDF) [file pgen.1002768.s004.pdf]

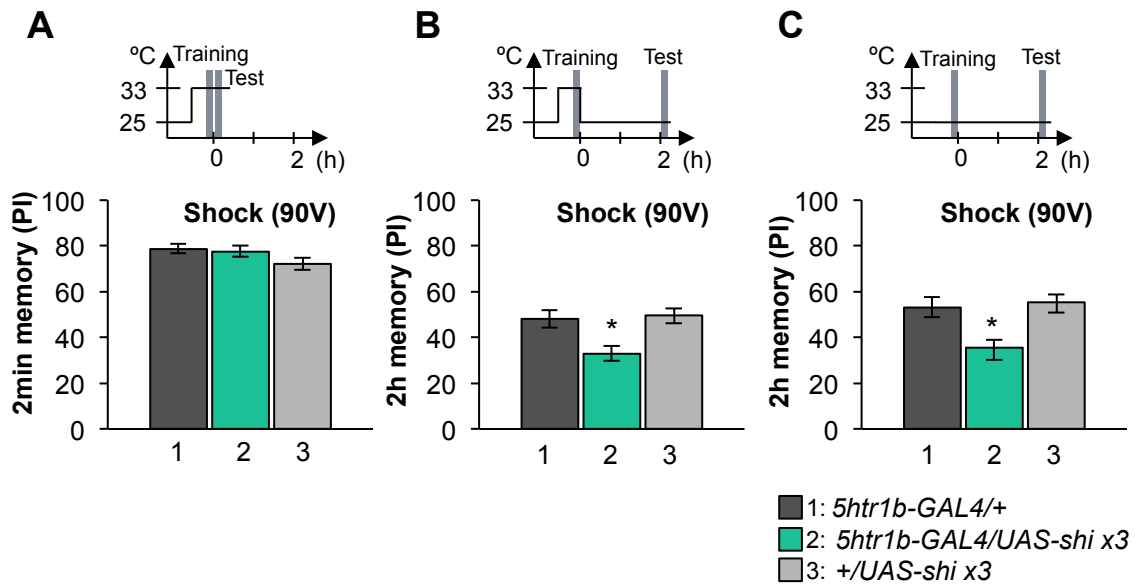

Aso et al., Figure S5

Supplement: Figure S5 — Multiple copies of UAS-shits1 preferentially impairs the acquisition of 2-hour memory in 5htr1b-GAL4. (A) Immediate memory of 5htr1b-GAL4/UAS-shix3 (multiple copies of UAS-shits1) is indistinguishable from the control groups. n = 14–16. (B) 2-h memory of 5htr1b-GAL4/UAS-shix3 is significantly affected. n = 16. (C) 5htr1b-GAL4/UAS-shix3 also show memory impairment at permissive temperature presumably due to the leaky effect of Shits1 at high expression level (see also Figure 9A). n = 12. (PDF) [file pgen.1002768.s005.pdf]

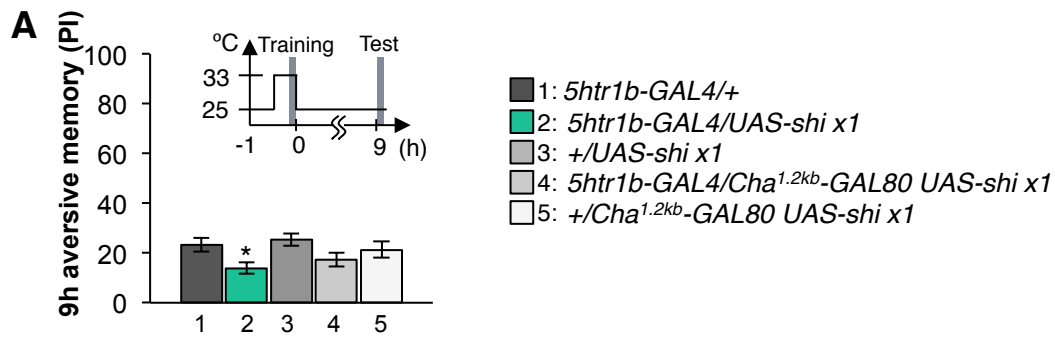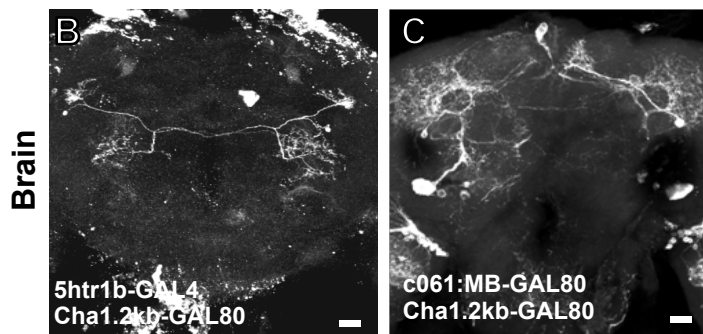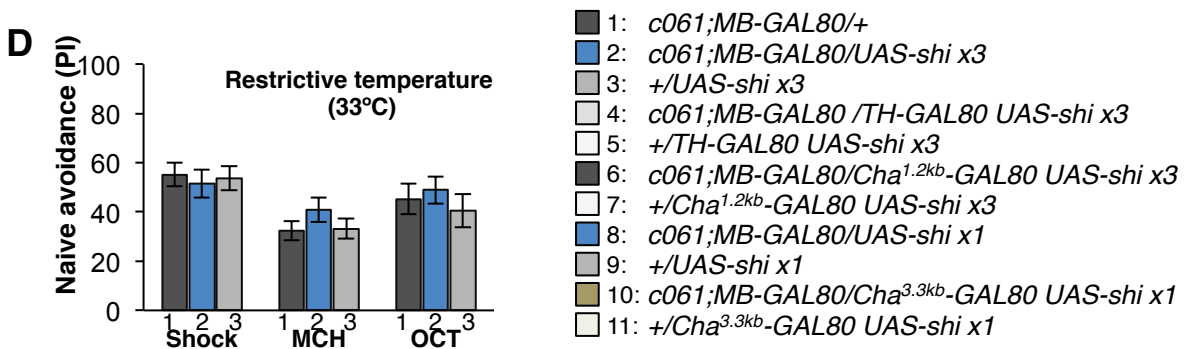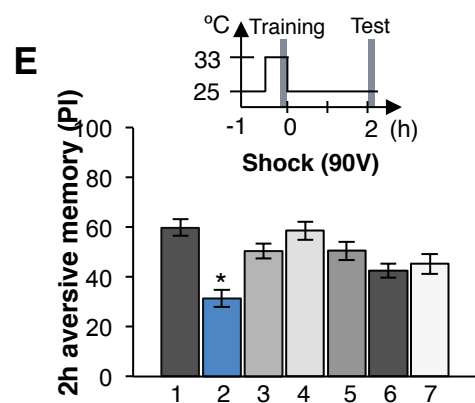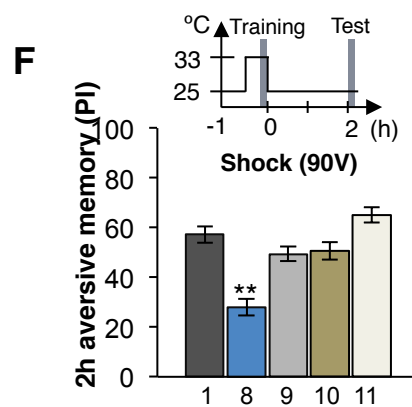

Aso et al., Figure S6

Supplement: Figure S6 — Requirement of MB-MV1/V1 and MB-MP1. (A) With Cha1.2kb-GAL80 that silence expression in both MB-MV1 and MB-V1, 5htr1b-GAL4/UAS-shits1does not show significant impairment of 9-hour memory (n = 20–28). (B–C) mCD8::GFP signals in the central brains with respective drivers (frontal view; dorsal up). Cha1.2kb-GAL80 silences expression in the PPL1 cluster cells in 5htr1b-GAL4 and c061. Scale bars represent 20 µm. (D) Blocking MB-MP1 neurons with c061;MB-GAL80 does not impair avoidance of electric shock, MCH or OCT (n = 12). (E) TH-GAL80 or Cha1.2kb-GAL80, c061;MB-GAL80/UAS- shits1does not show a significant memory impairment at 2 hour retention (n = 20–26). (D) The block with c061;MB-GAL80 driving a single copy of UAS- shits1 transgene results in a similar impairment of 2 hour memory, which is restored with Cha3.3kb-GAL80 (n = 12). See [25] for the expression pattern of c061;MB-GAL80 in combination with Cha3.3kb-GAL80. Bars and error bars represent the mean and s.e.m., respectively. * P<0.05; ** P<0.01; n.s. not significant. (PDF) [file pgen.1002768.s006.pdf]

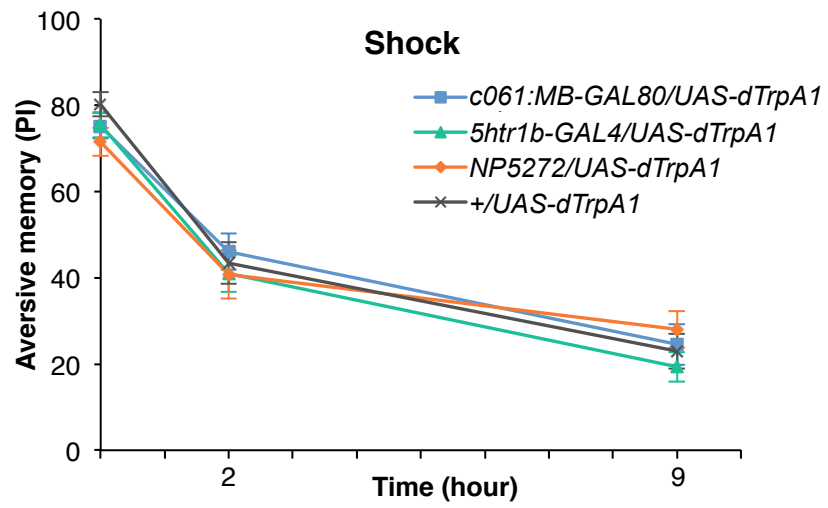

**Aso et al., Figure S7**

Supplement: Figure S7 — No significant effect of dTrpA1 expression in dopamine neurons at permissive temperature. At permissive temperature (25°C), flies expressing dTrpA1 with NP5272, 5htr1b-GAL4, c061;MB-GAL80 or no driver show indistinguishable levels of shock-induced memories at all the tested time points. n = 10–14. (PDF) [file pgen.1002768.s007.pdf]
