# Supplementary material for: Three Dopamine Pathways Induce Aversive Odor Memories with Different Stability
Source: PLoS Genet. 2012 Jul 12;8(7):e1002768. doi: 10.1371/journal.pgen.1002768 (PMC3395599; doi:10.1371/journal.pgen.1002768)
Supplement: Table S1 — List of crosses for behavior experiments. (DOC) [file pgen.1002768.s008.doc]

Supplementary Table S1: List of crosses for behavior experiments

| Figure | Males | | Females | |  |
| --- | --- | --- | --- | --- | --- |
| Figure 1U | *w;;TH-GAL4* | | *w* | |  |
|  | *w;;TH-GAL4* | | *w; UAS-dTrpA1* | |  |
|  | *w;;TH-GAL4* | | *w; UAS-dTrpA1; Cha^3.3kb^-GAL80* | |  |
|  | *w* | | *w; UAS-dTrpA1* | |  |
|  | *w* | | *w; UAS-dTrpA1; Cha^3.3kb^-GAL80* | |  |
| Figure 1V | *w;; DDC-GAL4 (HL8)* | | *w* | |  |
|  | *w;; DDC-GAL4 (HL8)* | | *w; UAS-dTrpA1* | |  |
|  | *w* | | *w; UAS-dTrpA1* | |  |
| Figure 1W | *w;; DDC-GAL4 (HL9)* | | *w* | |  |
|  | *w;; DDC-GAL4 (HL9)* | | *w; UAS-dTrpA1* | |  |
|  | *w* | | *w; UAS-dTrpA1* | |  |
| Figure 3Q | *w;MB-GAL80/CyO;NP0047/TM6b* | | *w* | |  |
|  | *w;MB-GAL80/CyO;NP0047/TM6b* | | *w; UAS-dTrpA1* | |  |
|  | *w;MB-GAL80/CyO;NP0047/TM6b* | | *w; TH-GAL80 UAS-dTrpA1* | |  |
|  | *w* | | *w; UAS-dTrpA1* | |  |
|  | *w* | | *w; TH-GAL80 UAS-dTrpA1* | |  |
| Figure 3R | *w;MB-GAL80/CyO;c259/TM6b* | | *w* | |  |
|  | *w;MB-GAL80/CyO;c259/TM6b* | | *w; UAS-dTrpA1* | |  |
|  | *w;MB-GAL80/CyO;c259/TM6b* | | *w; TH-GAL80 UAS-dTrpA1* | |  |
|  | *w* | | *w; UAS-dTrpA1* | |  |
|  | *w* | | *w; TH-GAL80 UAS-dTrpA1* | |  |
| Figure 4P | *w; 5htr1b-GAL4* | | *w* | |  |
|  | *w; 5htr1b-GAL4* | | *w; UAS-dTrpA1* | |  |
|  | *w; 5htr1b-GAL4* | | *w; TH-GAL80 UAS-dTrpA1* | |  |
|  | *w* | | *w; UAS-dTrpA1* | |  |
|  | *w* | | *w; TH-GAL80 UAS-dTrpA1* | |  |
| Figure 4Q | *w; 5htr1b-GAL4* | | *w* | |  |
|  | *w; 5htr1b-GAL4* | | *w; UAS-dTrpA1* | |  |
|  | *w; 5htr1b-GAL4* | | *w; UAS-dTrpA1; Cha^3.3kb^-GAL80* | |  |
|  | *w* | | *w; UAS-dTrpA1* | |  |
|  | *w* | | *w; UAS-dTrpA1; Cha^3.3kb^-GAL80* | |  |
|  | *w* | | *w* | |  |
| Figure 4R | *yw; NP7187* | | *w* | |  |
|  | *yw; NP7187* | | *w; UAS-dTrpA1* | |  |
|  | *w* | | *w; UAS-dTrpA1* | |  |
| Figure 5I | *w; NP7323* | | *w* | |  |
|  | *w; NP7323* | | *w; UAS-dTrpA1* | |  |
|  | *w* | | *w; UAS-dTrpA1* | |  |
| Figure 5J | *w; MZ19; Cha^3.3kb^-GAL80* | | *w* | |  |
|  | *w; MZ19; Cha^3.3kb^-GAL80* | | *w; UAS-dTrpA1* | |  |
|  | *w* | | *w; UAS-dTrpA1* | |  |
| Figure 8A | *yw;NP5272* | | *w* | |  |
|  | *yw;NP5272* | | *w UAS-Shi^ts1^;;UAS-Shi^ts1^ (multiple insertion)* | |  |
|  | *w* | | *w UAS-Shi^ts1^;;UAS-Shi^ts1^ (multiple insertion)* | |  |
| Figure 8B, 9A-D | *w; 5htr1b-GAL4* | | *w* | |  |
|  | *w; 5htr1b-GAL4* | | *w ;;UAS-Shi^ts1^  (single insertion)* | |  |
|  | *w* | | *w ;;UAS-Shi^ts1^  (single insertion)* | |  |
| Figure 8C, 9E-H | *w* | | *w c061;MB-GAL80* | |  |
|  | *w UAS-Shi^ts1^;;UAS-Shi^ts1^ (multiple insertion)* | | *w c061;MB-GAL80* | |  |
|  | *w UAS-Shi^ts1^;;UAS-Shi^ts1^ (multiple insertion)* | | *w* | |  |
|  |  | |  | |  |
| Figure | | Males | | Females | |
| Figure 10, 11, S7 | | *yw;NP5272* | | *w; UAS-dTrpA1* | |
|  | | *w;5htr1b-GAL4* | | *w; UAS-dTrpA1* | |
|  | | *w; UAS-dTrpA1* | | *w c061;MB-GAL80* | |
|  | | *w* | | *w; UAS-dTrpA1* | |
| Figure 12A, 13A,E | | *w;MB-GAL80/CyO;NP0047/TM6b* | | *w; UAS-dTrpA1* | |
|  | | *w;MB-GAL80/CyO;NP0047/TM6b* | | *w; NP5272 UAS-dTrpA1/CyO* | |
| Figure 12B, 13B,F | | *w; UAS-dTrpA1* | | *w; 5htr1b-GAL4* | |
|  | | *w; NP5272 UAS-dTrpA1/CyO* | | *w; 5htr1b-GAL4* | |
| Figure 12C | | *w; MZ840* | | *w; UAS-dTrpA1* | |
|  | | *w; MZ840* | | *w; NP5272 UAS-dTrpA1* | |
|  | | *w* | | *w; NP5272 UAS-dTrpA1* | |
| Figure 12D, 13C,G | | *w; UAS-dTrpA1* | | *w c061;MB-GAL80* | |
|  | | *w; 5htr1b-GAL4* | | *w c061;MB-GAL80 UAS-dTrpA1/CyO* | |
| Figure 12E,F, 13D,H | | *w; 5htr1b-GAL4* | | *w; UAS-dTrpA1* | |
|  | | *w; 5htr1b-GAL4* | | *w c061;MB-GAL80 UAS-dTrpA1/CyO* | |
|  | | *w* | | *w c061;MB-GAL80 UAS-dTrpA1/CyO* | |
| Figure S4 | | *w;;TH-GAL4* | | *w* | |
|  | | *w;;TH-GAL4* | | *w;UAS-dTrpA1* | |
|  | | *w* | | *w;UAS-dTrpA1* | |
|  | | *w;;TH-GAL4* | | *w;dTrpA1^ins^* | |
|  | | *w;;TH-GAL4* | | *UAS-dTrpA1;dTrpA1^ins^* | |
|  | | *w* | | *UAS-dTrpA1;dTrpA1^ins^* | |
|  | | *w;;TH-GAL4 dTrpA1^ins^* | | *w;dTrpA1^ins^* | |
|  | | *w;;TH-GAL4 dTrpA1^ins^* | | *UAS-dTrpA1;dTrpA1^ins^* | |
|  | | *w;; dTrpA1^ins^* | | *UAS-dTrpA1;dTrpA1^ins^* | |
| Figure S5 | | *w; 5htr1b-GAL4* | | *w* | |
|  | | *w; 5htr1b-GAL4* | | *w UAS-Shi^ts1^;;UAS-Shi^ts1^ (multiple insertion)* | |
|  | | *w* | | *w UAS-Shi^ts1^;;UAS-Shi^ts1^ (multiple insertion)* | |
| Figure S6A | | *w; 5htr1b-GAL4* | | *w* | |
|  | | *w; 5htr1b-GAL4* | | *w ;;UAS-Shi^ts1^  (single insertion)* | |
|  | | *w* | | *w ;;UAS-Shi^ts1^  (single insertion)* | |
|  | | *w; 5htr1b-GAL4* | | *w ;Cha^1.2kb^-GAL80; UAS-Shi^ts1^  (single insertion)* | |
|  | | *w* | | *w ;Cha^1.2kb^-GAL80; UAS-Shi^ts1^  (single insertion)* | |
| Figure S6D-F | | *w* | | *w c061;MB-GAL80* | |
|  | | *w UAS-Shi^ts1^;;UAS-Shi^ts1^ (multiple insertion)* | | *w c061;MB-GAL80* | |
|  | | *w UAS-Shi^ts1^;;UAS-Shi^ts1^ (multiple insertion)* | | *w* | |
|  | | *w UAS-Shi^ts1^;TH-GAL80/CyO;UAS-Shi^ts1^/TM6b* | | *w c061;MB-GAL80* | |
|  | | *w UAS-Shi^ts1^;TH-GAL80/CyO;UAS-Shi^ts1^/TM6b* | | *w* | |
|  | | *w UAS-Shi^ts1^;Cha^1.2kb^-GAL80/CyO;UAS-Shi^ts1^/TM6b* | | *w c061;MB-GAL80* | |
|  | | *w UAS-Shi^ts1^;Cha^1.2kb^-GAL80/CyO;UAS-Shi^ts1^/TM6b* | | *w* | |
|  | | *w ;;UAS-Shi^ts1^  (single insertion)* | | *w c061;MB-GAL80* | |
|  | | *w ;;UAS-Shi^ts1^  (single insertion)* | | *w* | |
|  | | *w;Cha^3.3kb^-GAL80 UAS-Shi^ts1^/TM6b* | | *w c061;MB-GAL80* | |
|  | | *w;Cha^3.3kb^-GAL80 UAS-Shi^ts1^/TM6b* | | *w* | |

References: *NP0047* (III), *NP7187* (X), *NP7323* (II) *NP5272* (II), *MZ840* (III) ;*c061* (X) ; *DDC-GAL4* (III) ; *HL9-GAL4* (III) ; *c259* (III) (http://www.fly-trap.org/); *MZ19;* (II) ; *UAS-dTrpA1(II)* ; *TH-GAL80 (II)* ; *Cha^3.3kb^-GAL80 (II, III)* .

**Supplemental References**

56. Young JM, Armstrong JD (2010) Structure of the adult central complex in Drosophila: organization of distinct neuronal subsets. J Comp Neurol 518: 1500-1524.

57. Ito K, Suzuki K, Estes P, Ramaswami M, Yamamoto D, et al. (1998) The organization of extrinsic neurons and their implications in the functional roles of the mushroom bodies in Drosophila melanogaster Meigen. Learn Mem 5: 52-77.

58. Kitamoto T (2002) Conditional disruption of synaptic transmission induces male-male courtship behavior in Drosophila. Proc Natl Acad Sci U S A 99: 13232-13237.
